# Mean almost periodicity and moment exponential stability of semi-discrete random cellular neural networks with fuzzy operations

**DOI:** 10.1371/journal.pone.0220861

**Published:** 2019-08-07

**Authors:** Sufang Han, Guoxin Liu, Tianwei Zhang

**Affiliations:** 1 School of Mathematics and Statistics, Central South University, Changsha, China; 2 City College, Kunming University of Science and Technology, Kunming, China; Lanzhou University of Technology, CHINA

## Abstract

By using the semi-discretization technique of differential equations, the discrete analogue of a kind of cellular neural networks with stochastic perturbations and fuzzy operations is formulated, which gives a more accurate characterization for continuous-time models than that by Euler scheme. Firstly, the existence of at least one *p*-th mean almost periodic sequence solution of the semi-discrete stochastic models with almost periodic coefficients is investigated by using Minkowski inequality, Hölder inequality and Krasnoselskii’s fixed point theorem. Secondly, the *p*-th moment global exponential stability of the semi-discrete stochastic models is also studied by using some analytical skills and the proof of contradiction. Finally, a problem of stochastic stabilization for discrete cellular neural networks is studied.

## Introduction

Cellular neural networks (CNNs) [1] have been widely applied in psychophysics, parallel computing, perception, robotics associative memory, image processing pattern recognition and combinatorial optimization. Most of these applications heavily depend on the (almost) periodicity and global exponential stability. Specifically, there are many scholars focusing on the study of the equilibrium points, (almost) periodic solutions and global exponential stability of CNNs with time delays in literatures [2–7]. For instance, Xu [7] considered the following CNNs with time delays:

$$\begin{array}{c} \frac{dx_{i}\left(t\right)}{dt}=-a_{i}\left(t\right)x_{i}\left(t\right)+\sum_{j=1}^{n}b_{ij}\left(t\right)f_{j}\left(x_{j}\left(t\right)\right)+\sum_{j=1}^{n}c_{ij}\left(t\right)g_{j}\left(x_{j}\left(t-\tau_{ij}\left(t\right)\right)\right)+I_{i}\left(t\right), \end{array}$$ (1)

where *n* denotes the number of units in a neural network, *x*_*i*_(*t*) corresponds to the state of the ith unit at time *t*, *a*_*i*_ > 0 represents the passive decay rates at time *t*, *f*_*j*_ and *g*_*j*_ are the neuronal output signal functions, *b*_*ij*_(*t*) and *c*_*ij*_(*t*) denote the strength of the *j*th unit on the *i*th unit at time *t*, *I*_*i*_(*t*) denotes the external inputs at time *t*, the continuous function *τ*_*ij*_(*t*) corresponds to the transmission delay at time *t*, *i*, *j* = 1, 2, …, *n*. In [7], the author studied the existence and exponential stability of anti-periodic solutions of system (1).

In real world applications, most of the problems are uncertain. They should be described by uncertain models and studied by using the research techniques for uncertain models. Stochastic and fuzzy theories are the most general and practical techniques for the research of uncertain models. On one hand, in the actual situations, uncertainties have a consequence on the performance of neural networks. The connection weights of the neurons depend on certain resistance and capacitance values that include modeling errors or uncertainties during the parameter identification process. Therefore, many neural network models described by stochastic differential equations [8, 9] have been widely studied over the last two decades, see [10–17]. On the other hand, fuzzy theory was conceived in the 1960s by L.A. Zadeh, it took about 20 years until the broader use of this theory in practice. Fuzzy technology joined forces with artificial neural networks and genetic algorithms under the title of computational intelligence or soft computing. In recent years, the research on the dynamical behaviours of fuzzy neural networks has attracted much attention, see [18–22]. To summarize, we consider the following CNNs with stochastic perturbations and fuzzy operations:

$$\begin{array}{ccc} dx_{i}\left(t\right) & = & [-a_{i}\left(t\right)x_{i}\left(t\right)\\ & & +\sum_{j=1}^{n}b_{ij}\left(t\right)f_{j}\left(x_{j}\left(t\right)\right)+\sum_{j=1}^{n}c_{ij}\left(t\right)g_{j}\left(x_{j}\left(t-\tau_{ij}\left(t\right)\right)\right)+\overset{n}{\underset{j=1}{⋀}}\alpha_{ij}g_{j}\left(x_{j}\left(t-\tau_{ij}\left(t\right)\right)\right)\\ & & +\overset{n}{\underset{j=1}{⋀}}T_{ij}\mu_{j}+\overset{n}{\underset{j=1}{⋁}}\beta_{ij}g_{j}\left(x_{j}\left(t-\tau_{ij}\left(t\right)\right)\right)+\overset{n}{\underset{j=1}{⋁}}S_{ij}\mu_{j}+I_{i}\left(t\right)]dt\\ & & +\sum_{j=1}^{n}d_{ij}\left(t\right)\sigma_{j}\left(x_{j}\left(t-\eta_{ij}\left(t\right)\right)\right)dw_{j}\left(t\right), \end{array}$$ (2)

where *α*_*ij*_, *β*_*ij*_, *T*_*ij*_ and *S*_*ij*_ are elements of fuzzy feedback MIN, MAX template, fuzzy feed forward MIN and MAX template, respectively; ⋀ and ⋁ denote the fuzzy AND and fuzzy OR operation, respectively; *d*_*ij*_, *η*_*ij*_ and *σ*_*j*_ are similarly specified as that in system (1), *w*_*j*_ is the standard Brownian motion defined on a complete probability space, *i*, *j* = 1, 2, …, *n*.

Periodicity often appears in implicit ways in various natural phenomena. Though one can deliberately periodically fluctuate environmental parameters in laboratory experiments, fluctuations in nature are hardly periodic. Almost periodicity is more likely to accurately describe natural fluctuations [23–30]. The concept of mean almost periodicity is important in probability especially for investigations on stochastic processes. In particular, mean almost periodicity enables us to understand the impact of the noise or stochastic perturbation on the corresponding recurrent motions, is of great concern in the study of stochastic differential equations and random dynamical systems. The notion of almost periodic stochastic process was proposed in the 1980s and since then almost periodic solutions to stochastic differential equations driven have been studied by many authors. On the other hand, the problem of stability analysis of dynamic systems has a rich, long history of literature [31–35]. All the applications of such stochastic dynamical systems depend on qualitative behavior such as stability, existence and uniqueness, convergence and so on. In particular, exponential stability is a significant one in the design and applications of neural networks. Therefore, the mean almost periodicity and moment exponential stability of various kinds of stochastic neural networks has been reported in [36–41].

The discrete-time neural networks become more important than the continuous-time counterparts when implementing the neural networks in a digital way. In order to investigate the dynamical characteristics with respect to digital signal transmission, it is essential to formulate the discrete analog of neural networks. A large number of literatures have been obtained for the dynamics of discrete-time neural networks formulated by Euler scheme [42–46]. Mohamad and Gopalsamy [47, 48] proposed a novel method (i.e., semi-discretization technique) in formulating a discrete-time analogue of the continuous-time neural networks, which faithfully preserved the characteristics of their continuous-time counterparts. In [47], the authors employed computer simulations to show that semi-discrete models give a more accurate characterization for the corresponding continuous-time models than that by Euler scheme. With the help of the semi-discretization technique [47], many scholars obtained the semi-discrete analogue of the continuous-time neural networks and some meaningful results were gained for the dynamic behaviours of the semi-discrete neural networks, such as periodic solutions, almost periodic solutions and global exponential stability, see [49–55]. For instance, Huang et al. [52] discussed the following semi-discrete cellular neural networks:

$$\begin{array}{c} x_{i}\left(k+1\right)=e^{-a_{i}\left(k\right)}x_{i}\left(k\right)+\frac{1-e^{-a_{i}\left(k\right)}}{a_{i}\left(k\right)}[\sum_{j=1}^{n}b_{ij}\left(k\right)f_{j}\left(x_{j}\left(k\right)\right)+I_{i}\left(k\right)], \end{array}$$ (3)

where k∈Z, Z denotes the set of integers, *i* = 1, 2, …, *n*. In [52], sufficient conditions were obtained for the existence of a unique stable almost periodic sequence solution of system (3) under assumption of almost periodicity of coefficients of system (3). Similarly, Ji [55] considered a kind of semi-discrete Cohen-Grossberg neural networks with delays and the same problems as that in [52] were studied. In 2014, by using semi-discretization technique [47], Huang et al. [53] obtained the following semi-discrete models for a class of general neural networks:

$$\begin{array}{c} x_{i}\left(k+1\right)=e^{-a_{i}\left(k\right)}x_{i}\left(k\right)+\frac{1-e^{-a_{i}\left(k\right)}}{a_{i}\left(k\right)}[\sum_{l=1}^{m}\sum_{j=1}^{n}b_{ijl}\left(k\right)f_{j}\left(x_{j}\left(k-\tau_{ijl}\right)\right)+I_{i}\left(k\right)], \end{array}$$ (4)

where k∈Z, *i* = 1, 2, …, *n*. The authors [53] derived the existence of locally exponentially convergent 2^*N*^ almost periodic sequence solutions of system (4). Kong and Fang [50] in 2018 investigated a class of semi-discrete neutral-type neural networks with delays and some results are acquired for the existence of a unique pseudo almost periodic sequence solution which is globally attractive and globally exponentially stable.

However, the disquisitive models in literatures [49–55] are deterministic. Stimulated by this point, we should consider random factors in the studied models, such as system (2). By using the semi-discretization technique [47], Krasnoselskii’s fixed point theorem and stochastic theory, the main aim of this paper is to establish some decision theorems for the existence of *p*-th mean almost periodic sequence solutions and *p*-th moment global exponential stability for the semi-discrete analogue of uncertain system (2). The work of this paper is a continuation of that in [52–55] and the results in this paper complement the corresponding results in [52–55]. The main contributions of this paper are summed up as: **(1)** The semi-discrete analogue is established for stochastic fuzzy CNNs (2); **(2)** A Volterra additive equation is derived for the solution of the semi-discrete stochastic fuzzy CNNs; **(3)** The existence of *p*-th mean almost periodic sequence solutions is obtained; **(4)** A decision theorem is acquired for the *p*-th moment global exponential stability; **(5)** A problem of stochastic stabilization for discrete CNNs is proposed and researched.

Throughout this paper, we use the following notations. Let R denote the set of real numbers. $R^{n}$ denotes the *n*-dimensional real vector space. Let (Ω,F,P) be a complete probability space. Denote by $BC(Z,L^{p}\left(\Omega ,R^{n}\right))$ the vector space of all bounded continuous functions from Z to $L^{p}\left(\Omega ,R^{n}\right)$, where $L^{p}\left(\Omega ,R^{n}\right)$ denotes the collection of all *p*-th integrable $R^{n}$-valued random variables. Then $BC(Z,L^{p}\left(\Omega ,R^{n}\right))$ is a Banach space with the norm ${\left\|X\right\|}_{p}={\text{sup}}_{k\in Z}{\left|X\right|}_{p}$, ${\left|X\right|}_{p}={\text{max}}_{1\leq i\leq n}\left(E\right|x_{i}\left(k\right){{|}^{p})}^{\frac{1}{p}}$, $\forall X=\left\{x_{i}\right\}≔{\left(x_{1},x_{2},\ldots ,x_{n}\right)}^{T}\in BC\left(Z,L^{p}\left(\Omega ,R^{n}\right)\right)$, where *p* > 1 and *E*(⋅) stands for the expectation operator with respect to the given probability measure *P*. Set $\bar{f}=\underset{k\in Z}{\sup}\left|f\left(k\right)\right|$ and $\underline{f}=\underset{k\in Z}{\inf}\left|f\left(k\right)\right|$ for bounded real function *f* defined on Z. ${\left[a,b\right]}_{Z}=\left[a,b\right]\cap Z$, ∀a,b∈R.

## Discrete analogue and preliminaries

### The semi-discretization model

For the sake of gaining the discrete analogue of system (2) with the semi-discretization technique [47], the following uncertain CNNs with piecewise constant arguments corresponding to system (2) have been taken into account:

$$\begin{array}{ccc} dx_{i}\left(t\right) & = & [-a_{i}\left(\left[t\right]\right)x_{i}\left(t\right)+\sum_{j=1}^{n}b_{ij}\left(\left[t\right]\right)f_{j}\left(x_{j}\left(\left[t\right]\right)\right)+\sum_{j=1}^{n}c_{ij}\left(\left[t\right]\right)g_{j}\left(x_{j}\left(\left[t\right]-\tau_{ij}\left(\left[t\right]\right)\right)\right)\\ & & +\overset{n}{\underset{j=1}{⋀}}\alpha_{ij}g_{j}\left(x_{j}\left(\left[t\right]-\tau_{ij}\left(\left[t\right]\right)\right)\right)+\overset{n}{\underset{j=1}{⋀}}T_{ij}\mu_{j}+\overset{n}{\underset{j=1}{⋁}}\beta_{ij}g_{j}\left(x_{j}\left(\left[t\right]-\tau_{ij}\left(\left[t\right]\right)\right)\right)\\ & & +\overset{n}{\underset{j=1}{⋁}}S_{ij}\mu_{j}+\sum_{j=1}^{n}d_{ij}\left(\left[t\right]\right)\sigma_{j}\left(x_{j}\left(\left[t\right]-\eta_{ij}\left(\left[t\right]\right)\right)\right)\Delta w_{j}\left(\left[t\right]\right)+I_{i}\left(\left[t\right]\right)]dt, \end{array}$$

where [*t*] denotes the integer part of *t*, *i* = 1, 2, …, *n*. Here the discrete analogue of the stochastic parts of system (2) is obtained by Euler scheme, i.e., d*w*_*j*_(*t*) = Δ*w*_*j*_([*t*])d*t* = [*w*_*j*_([*t*] + 1) − *w*_*j*_([*t*])]d*t*, *j* = 1, 2, …, *n*. For each *t*, there exists an integer *k* such that *k* ≤ *t* < *k* + 1. Then the above equation becomes

$$\begin{array}{ccc} dx_{i}\left(t\right) & = & [-a_{i}\left(k\right)x_{i}\left(t\right)+\sum_{j=1}^{n}b_{ij}\left(k\right)f_{j}\left(x_{j}\left(k\right)\right)+\sum_{j=1}^{n}c_{ij}\left(k\right)g_{j}\left(x_{j}\left(k-\tau_{ij}\left(k\right)\right)\right)\\ & & +\overset{n}{\underset{j=1}{⋀}}\alpha_{ij}g_{j}\left(x_{j}\left(k-\tau_{ij}\left(k\right)\right)\right)+\overset{n}{\underset{j=1}{⋀}}T_{ij}\mu_{j}+\overset{n}{\underset{j=1}{⋁}}\beta_{ij}g_{j}\left(x_{j}\left(k-\tau_{ij}\left(k\right)\right)\right)\\ & & +\overset{n}{\underset{j=1}{⋁}}S_{ij}\mu_{j}+\sum_{j=1}^{n}d_{ij}\left(k\right)\sigma_{j}\left(x_{j}\left(k-\eta_{ij}\left(k\right)\right)\right)\Delta w_{j}\left(k\right)+I_{i}\left(k\right)]dt, \end{array}$$

where *i* = 1, 2, …, *n*. Integrating the above equation from *k* to *t* and letting *t* → *k* + 1, we achieve the discrete analogue of system (2) as follows:

$$\begin{array}{ccc} x_{i}\left(k+1\right) & = & e^{-a_{i}\left(k\right)}x_{i}\left(k\right)\\ & & +\frac{1-e^{-a_{i}\left(k\right)}}{a_{i}\left(k\right)}[\sum_{j=1}^{n}b_{ij}\left(k\right)f_{j}\left(x_{j}\left(k\right)\right)+\sum_{j=1}^{n}c_{ij}\left(k\right)g_{j}\left(x_{j}\left(k-\tau_{ij}\left(k\right)\right)\right)\\ & & +\overset{n}{\underset{j=1}{⋀}}\alpha_{ij}g_{j}\left(x_{j}\left(k-\tau_{ij}\left(k\right)\right)\right)+\overset{n}{\underset{j=1}{⋀}}T_{ij}\mu_{j}+\overset{n}{\underset{j=1}{⋁}}\beta_{ij}g_{j}\left(x_{j}\left(k-\tau_{ij}\left(k\right)\right)\right)\\ & & +\overset{n}{\underset{j=1}{⋁}}S_{ij}\mu_{j}+\sum_{j=1}^{n}d_{ij}\left(k\right)\sigma_{j}\left(x_{j}\left(k-\eta_{ij}\left(k\right)\right)\right)\Delta w_{j}\left(k\right)+I_{i}\left(k\right)], \end{array}$$ (5)

where k∈Z, *i* = 1, 2, …, *n*.

### Volterra additive equation for the solution of system (5)

**Lemma 1**. *X* = {*x*_*i*_} is a solution of system (5) if and only if

$$\begin{array}{ccc} x_{i}\left(k\right) & = & \prod_{s=k_{0}}^{k-1}e^{-a_{i}\left(s\right)}x_{i}\left(k_{0}\right)+\sum_{v=k_{0}}^{k-1}\prod_{s=v+1}^{k-1}\frac{e^{-a_{i}\left(s\right)}\left[1-e^{-a_{i}\left(v\right)}\right]}{a_{i}\left(v\right)}[\sum_{j=1}^{n}b_{ij}\left(v\right)f_{j}\left(x_{j}\left(v\right)\right)\\ & & +\sum_{j=1}^{n}c_{ij}\left(v\right)g_{j}\left(x_{j}\left(v-\tau_{ij}\left(v\right)\right)\right)+\overset{n}{\underset{j=1}{⋀}}\alpha_{ij}g_{j}\left(x_{j}\left(v-\tau_{ij}\left(v\right)\right)\right)\\ & & +\overset{n}{\underset{j=1}{⋀}}T_{ij}\mu_{j}+\overset{n}{\underset{j=1}{⋁}}\beta_{ij}g_{j}\left(x_{j}\left(v-\tau_{ij}\left(v\right)\right)\right)+\overset{n}{\underset{j=1}{⋁}}S_{ij}\mu_{j}\\ & & +\sum_{j=1}^{n}d_{ij}\left(v\right)\sigma_{j}\left(x_{j}\left(v-\eta_{ij}\left(v\right)\right)\right)\Delta w_{j}\left(v\right)+I_{i}\left(v\right)], \end{array}$$ (6)

where $k_{0}\in Z$, $k\in {\left[k_{0}+1,+\infty \right)}_{Z}$, *i* = 1, 2, …, *n*.

*Proof*. Let

$$\begin{array}{ccc} & & F_{i}\left(k,x\right)\\ & ≔ & \sum_{j=1}^{n}b_{ij}\left(k\right)f_{j}\left(x_{j}\left(k\right)\right)+\sum_{j=1}^{n}c_{ij}\left(k\right)g_{j}\left(x_{j}\left(k-\tau_{ij}\left(k\right)\right)\right)\\ & & +\overset{n}{\underset{j=1}{⋀}}\alpha_{ij}g_{j}\left(x_{j}\left(k-\tau_{ij}\left(k\right)\right)\right)+\overset{n}{\underset{j=1}{⋀}}T_{ij}\mu_{j}+\overset{n}{\underset{j=1}{⋁}}\beta_{ij}g_{j}\left(x_{j}\left(k-\tau_{ij}\left(k\right)\right)\right)+\overset{n}{\underset{j=1}{⋁}}S_{ij}\mu_{j}\\ & & +\sum_{j=1}^{n}d_{ij}\left(k\right)\sigma_{j}\left(x_{j}\left(k-\eta_{ij}\left(k\right)\right)\right)\Delta w_{j}\left(k\right)+I_{i}\left(k\right),\;k\in Z,\;\;i=1,2,\ldots ,n. \end{array}$$

Assume that *X* = {*x*_*i*_} is a solution of system (5). By Δ[*u*(*k*)*v*(*k*)] = [Δ*u*(*k*)]*v*(*k*) + *u*(*k* + 1)[Δ*v*(*k*)] and system (5), it gets

$$\begin{array}{c} \Delta [\prod_{s=0}^{k-1}e^{a_{i}\left(s\right)}x_{i}\left(k\right)]=\prod_{s=0}^{k}\frac{e^{a_{i}\left(s\right)}\left[1-e^{-a_{i}\left(k\right)}\right]}{a_{i}\left(k\right)}F_{i}\left(k,x\right),\;k\in Z,\;\;i=1,2,\ldots ,n. \end{array}$$

So

$$\sum_{v=k_{0}}^{k-1}\Delta [\prod_{s=0}^{v-1}e^{a_{i}\left(s\right)}x_{i}\left(v\right)]=\sum_{v=k_{0}}^{k-1}\prod_{s=0}^{v}\frac{e^{a_{i}\left(s\right)}\left[1-e^{-a_{i}\left(v\right)}\right]}{a_{i}\left(v\right)}F_{i}\left(v,x\right)$$

is equivalent to

$$\prod_{s=0}^{k-1}e^{a_{i}\left(s\right)}x_{i}\left(k\right)=\prod_{s=0}^{k_{0}-1}e^{a_{i}\left(s\right)}x_{i}\left(k_{0}\right)+\sum_{v=k_{0}}^{k-1}\prod_{s=0}^{v}\frac{e^{a_{i}\left(s\right)}\left[1-e^{-a_{i}\left(v\right)}\right]}{a_{i}\left(v\right)}F_{i}\left(v,x\right),$$

where *i* = 1, 2, …, *n*, k∈Z. By the above equations, we can easily derive (6).

If *X* = {*x*_*i*_} satisfies (6), then

$$x_{i}\left(k\right)=\prod_{s=k_{0}}^{k-1}e^{-a_{i}\left(s\right)}x_{i}\left(0\right)+\sum_{v=k_{0}}^{k-1}\prod_{s=v+1}^{k-1}\frac{e^{a_{i}\left(s\right)}\left[1-e^{-a_{i}\left(v\right)}\right]}{a_{i}\left(v\right)}F_{i}\left(v,x\right),$$

which implies that

$$\begin{array}{ccc} x_{i}\left(k+1\right) & = & \prod_{s=k_{0}}^{k}e^{-a_{i}\left(s\right)}x_{i}\left(0\right)+\sum_{v=k_{0}}^{k}\prod_{s=v+1}^{k}\frac{e^{a_{i}\left(s\right)}\left[1-e^{-a_{i}\left(v\right)}\right]}{a_{i}\left(v\right)}F_{i}\left(v,x\right)\\ & = & e^{-a_{i}\left(k\right)}[\prod_{s=k_{0}}^{k-1}e^{-a_{i}\left(s\right)}x_{i}\left(0\right)\\ & & +\sum_{v=k_{0}}^{k-1}\prod_{s=v+1}^{k-1}\frac{e^{a_{i}\left(s\right)}\left[1-e^{-a_{i}\left(v\right)}\right]}{a_{i}\left(v\right)}F_{i}\left(v,x\right)]+\frac{1-e^{-a_{i}\left(k\right)}}{a_{i}\left(k\right)}F_{i}\left(k,x\right)\\ & = & e^{-a_{i}\left(k\right)}x_{i}\left(k\right)+\frac{1-e^{-a_{i}\left(k\right)}}{a_{i}\left(k\right)}F_{i}\left(k,x\right), \end{array}$$

where *i* = 1, 2, …, *n*, k∈Z. Therefore, *X* = {*x*_*i*_} is a solution of system (5). This completes the proof.

### Some lemmas

**Lemma 2**. ([56]) (Minkowski inequality) Assume that *p* ≥ 1, *E*|*ξ*|^*p*^ < ∞, *E*|*η*|^*p*^ < ∞, then

$$\begin{array}{c} {\left(E|\xi +\eta \right|}^{p}{)}^{1/p}\leq {\left(E|\xi \right|}^{p}{)}^{1/p}+{\left(E|\eta \right|}^{p}{)}^{1/p}. \end{array}$$

**Lemma 3**. ([56]) (Hölder inequality) Assume that *p* > 1, then

$$\begin{array}{c} \sum_{k}\left|a_{k}b_{k}\right|\leq [\sum_{k}\left|a_{k}\right|{]}^{1-1/p}[\sum_{k}|a_{k}\left|\right|b_{k}{|}^{p}{]}^{1/p}. \end{array}$$

If *p* = 1, then ∑_*k*_|*a*_*k*_*b*_*k*_| ≤ (∑_*k*_|*a*_*k*_|)(sup_*k*_ |*b*_*k*_|).

**Lemma 4**. ([9]) Suppose that $g\in L^{2}\left(\left[a,b\right],R\right)$, then

$$\begin{array}{c} E[\underset{t\in [a,b]}{\text{sup}}|\int_{a}^{t}g\left(s\right)d\omega \left(s\right){|}^{p}]\leq C_{p}E[\int_{a}^{b}{\left|g\left(t\right)\right|}^{2}dt{]}^{\frac{p}{2}}, \end{array}$$

where

$$\begin{array}{c} C_{p}=\{\begin{array}{cc} {\left(32/p\right)}^{p/2},\; & 0<p<2,\\ 4,\; & p=2,\\ {}[\frac{p^{p+1}}{2{\left(p-1\right)}^{\left(p-1\right)}}{]}^{\frac{p}{2}},\; & p>2. \end{array} \end{array}$$

**Lemma 5**. Assume that {x(k):k∈Z} is real-valued stochastic process and *w*(*k*) is the standard Brownian motion, then

$$\begin{array}{c} E|x\left(k\right)\Delta w\left(k\right){|}^{p}\leq C_{p}E|x\left(k\right){|}^{p},\;k\in Z, \end{array}$$

where *C*_*p*_ is defined as that in Lemma 4, *p* > 0.

*Proof*. By Lemma 4, it follows that

$$\begin{array}{c} E|x\left(k\right)\Delta w\left(k\right){|}^{p}=E|\int_{k}^{k+1}x\left(k\right)\;dw\left(s\right){|}^{p}\leq C_{p}E|\int_{k}^{k+1}x^{2}\left(k\right)\;ds{|}^{\frac{p}{2}}\leq C_{p}E|x\left(k\right){|}^{p}, \end{array}$$

where k∈Z. This completes the proof.

**Lemma 6**. ([57]) Suppose *X* = {*x*_*i*_} and *Y* = {*y*_*i*_} are two states of system (5), then we have

$$\begin{array}{c} |\overset{n}{\underset{j=1}{⋀}}\alpha_{ij}f_{j}\left(x_{j}\right)-\overset{n}{\underset{j=1}{⋀}}\alpha_{ij}f_{j}\left(y_{j}\right)|\leq \sum_{j=1}^{n}|\alpha_{ij}\left|\right|f_{j}\left(x_{j}\right)-f_{j}\left(y_{j}\right)| \end{array}$$

and

$$\begin{array}{c} |\overset{n}{\underset{j=1}{⋁}}\beta_{ij}f_{j}\left(x_{j}\right)-\overset{n}{\underset{j=1}{⋁}}\beta_{ij}f_{j}\left(y_{j}\right)|\leq \sum_{j=1}^{n}|\beta_{ij}\left|\right|f_{j}\left(x_{j}\right)-f_{j}\left(y_{j}\right)|,\;i=1,2,\ldots ,n. \end{array}$$

## *p*-th mean almost periodic sequence solution

**Definition 1**. ([8]) A stochastic process $X\in BC(Z;L^{p}\left(\Omega ;R^{n}\right))$ is said to be *p*-th mean almost periodic sequence if for each *ϵ* > 0, there exists an integer *l*(*ϵ*) > 0 such that each interval of length *l*(*ϵ*) contains an integer *ω* for which

$$\begin{array}{c} {\left|X\left(k+\omega \right)-X\left(k\right)\right|}_{p}=\underset{1\leq i\leq n}{\text{max}}(E|x_{i}\left(k+\omega \right)-x_{i}{\left(k\right)|}^{p}{)}^{\frac{1}{p}}<\epsilon ,\;\forall k\in Z. \end{array}$$

A stochastic process *X*, which is 2-nd mean almost periodic sequence will be called square-mean almost periodic sequence. Like for classical almost periodic functions, the number *ω* will be called an *ϵ*-translation of *X*.

**Lemma 7**. ([58]) Assume that Λ is a closed convex nonempty subset of a Banach space X. Suppose further that B and C map Λ into X such that

1. B is continuous and BΛ is contained in a compact set,
2. *x*, *y* ∈ Λ implies that Bx+Cy∈Λ,
3. C is a contraction mapping.

Then there exists a *z* ∈ Λ such that z=Bz+Cz.

Throughout this paper, we always assume that the following conditions are satisfied:

(*H*_1_) ${\underline{a}}_{i}>0$, *i* = 1, 2, …, *n*.

(*H*_2_) There are several positive constants $L_{j}^{f}$, $L_{j}^{g}$ and $L_{j}^{\sigma }$ such that

$$\begin{array}{c} |f_{j}\left(u\right)-f_{j}\left(v\right)|\leq L_{j}^{f}\left|u-v\right|, \end{array}$$ (7)

$$\begin{array}{c} |g_{j}\left(u\right)-g_{j}\left(v\right)|\leq L_{j}^{g}\left|u-v\right|, \end{array}$$ (8)

$$\begin{array}{c} |\sigma_{j}\left(u\right)-\sigma_{j}\left(v\right)|\leq L_{j}^{\sigma }\left|u-v\right|, \end{array}$$ (9)

∀u,v∈R, where *j* = 1, 2, …, *n*.

Define

$$\begin{array}{c} \bar{a}≔\underset{1\leq i\leq n}{\text{max}}{\bar{a}}_{i},\;\underline{a}≔\min_{1\leq i\leq n}{\underline{a}}_{i},\;D^{*}≔\underset{1\leq i\leq n}{\text{max}}\sum_{j=1}^{n}\{{\bar{b}}_{ij}L_{j}^{f}+(|\alpha_{ij}\left|+\right|\beta_{ij}|+{\bar{c}}_{ij})L_{j}^{g}\}, \end{array}$$

$$\begin{array}{c} K^{*}≔\underset{1\leq i\leq n}{\text{max}}\sum_{j=1}^{n}{\bar{d}}_{ij}L_{j}^{\sigma },\;r_{p}≔\frac{\left(1-e^{-\bar{a}}\right)}{\underline{a}\left(1-e^{-\underline{a}}\right)}\{D^{*}+K^{*}C_{p}^{\frac{1}{p}}\},\;\beta_{p}≔\frac{\alpha_{p}}{1-r_{p}}, \end{array}$$

$$\begin{array}{ccc} \alpha_{p} & ≔ & \frac{\left(1-e^{-\bar{a}}\right)}{\underline{a}\left(1-e^{-\underline{a}}\right)}\underset{1\leq i\leq n}{\text{max}}[\sum_{j=1}^{n}({\bar{b}}_{ij}|f_{j}\left(0\right)|+{\bar{c}}_{ij}\left|g_{j}\left(0\right)\right|)\\ & & +\sum_{j=1}^{n}(|\alpha_{ij}\left|+\right|\beta_{ij}|)|g_{j}\left(0\right)|+\sum_{j=1}^{n}(|T_{ij}\left|+\right|S_{ij}|)\left|\mu_{j}\right|+{\bar{I}}_{i}+\sum_{j=1}^{n}{\bar{d}}_{ij}\sigma_{j}\left(0\right)C_{p}^{\frac{1}{p}}]. \end{array}$$

**Theorem 1**. *Assume that all coefficients in system* (5) *excluding the Brownian motions are almost periodic sequences*, (*H*_1_)-(*H*_2_) *hold and the following condition is satisfied*:

(*H*_3_) *r*_*p*_ < 1, *where p* > 1.

*Then there exists a p-th mean almost periodic sequence solution X of system* (5) *with* ‖*X*‖_*p*_ ≤ *β*_*p*_.

*Proof*. Let $\Lambda \subseteq BC(Z;L^{p}\left(\Omega ;R^{n}\right))$ be the collection of all *p*-th mean almost periodic sequences *X* = {*x*_*i*_} satisfying ‖*X*‖_*p*_ ≤ *β*_*p*_.

Firstly, *X* = {*x*_*i*_} is described by

$$\begin{array}{ccc} x_{i}\left(k\right) & = & \sum_{v=-\infty }^{k-1}\prod_{s=v+1}^{k-1}\frac{e^{-a_{i}\left(s\right)}\left[1-e^{-a_{i}\left(v\right)}\right]}{a_{i}\left(v\right)}[\sum_{j=1}^{n}b_{ij}\left(v\right)f_{j}\left(x_{j}\left(v\right)\right)\\ & & +\sum_{j=1}^{n}c_{ij}\left(v\right)g_{j}\left(x_{j}\left(v-\tau_{ij}\left(v\right)\right)\right)+\overset{n}{\underset{j=1}{⋀}}\alpha_{ij}g_{j}\left(x_{j}\left(v-\tau_{ij}\left(v\right)\right)\right)+\overset{n}{\underset{j=1}{⋀}}T_{ij}\mu_{j}\\ & & +\overset{n}{\underset{j=1}{⋁}}\beta_{ij}g_{j}\left(x_{j}\left(v-\tau_{ij}\left(v\right)\right)\right)+\overset{n}{\underset{j=1}{⋁}}S_{ij}\mu_{j}\\ & & +\sum_{j=1}^{n}d_{ij}\left(v\right)\sigma_{j}\left(x_{j}\left(v-\eta_{ij}\left(v\right)\right)\right)\Delta w_{j}\left(v\right)+I_{i}\left(v\right)], \end{array}$$ (10)

where *i* = 1, 2, …, *n*, k∈Z. Obviously, (10) is well defined and satisfies (6). So we define ΦX(k)=BX(k)+CX(k), where

$$\begin{array}{c} \Phi X\left(k\right)={\left({\left(\Phi X\right)}_{1}\left(k\right),{\left(\Phi X\right)}_{2}\left(k\right),\ldots ,{\left(\Phi X\right)}_{n}\left(k\right)\right)}^{T}, \end{array}$$

$$\begin{array}{c} {\left(\Phi X\right)}_{i}\left(k\right)={\left(BX\right)}_{i}\left(k\right)+{\left(CX\right)}_{i}\left(k\right), \end{array}$$ (11)

$$\begin{array}{ccc} {\left(BX\right)}_{i}\left(k\right) & = & \sum_{v=-\infty }^{k-1}\prod_{s=v+1}^{k-1}\frac{e^{-a_{i}\left(s\right)}\left[1-e^{-a_{i}\left(v\right)}\right]}{a_{i}\left(v\right)}[\sum_{j=1}^{n}b_{ij}\left(v\right)f_{j}\left(x_{j}\left(v\right)\right)\\ & & +\sum_{j=1}^{n}c_{ij}\left(v\right)g_{j}\left(x_{j}\left(v-\tau_{ij}\left(v\right)\right)\right)+\overset{n}{\underset{j=1}{⋀}}\alpha_{ij}g_{j}\left(x_{j}\left(v-\tau_{ij}\left(v\right)\right)\right)\\ & & +\overset{n}{\underset{j=1}{⋀}}T_{ij}\mu_{j}+\overset{n}{\underset{j=1}{⋁}}\beta_{ij}g_{j}\left(x_{j}\left(v-\tau_{ij}\left(v\right)\right)\right)\\ & & +\overset{n}{\underset{j=1}{⋁}}S_{ij}\mu_{j}+I_{i}\left(v\right)], \end{array}$$ (12)

$$\begin{array}{c} {\left(CX\right)}_{i}\left(k\right)=\sum_{v=-\infty }^{k-1}\prod_{s=v+1}^{k-1}\frac{e^{-a_{i}\left(s\right)}\left[1-e^{-a_{i}\left(v\right)}\right]}{a_{i}\left(v\right)}\sum_{j=1}^{n}d_{ij}\left(v\right)\sigma_{j}\left(x_{j}\left(v-\eta_{ij}\left(v\right)\right)\right)\Delta w_{j}\left(v\right), \end{array}$$ (13)

where *i* = 1, 2, …, *n*, k∈Z.

Let $X^{0}=\left\{x_{i}^{0}\right\}$ be defined as

$$\begin{array}{ccc} x_{i}^{0}\left(k\right) & = & \sum_{v=-\infty }^{k-1}\prod_{s=v+1}^{k-1}\frac{e^{-a_{i}\left(s\right)}\left[1-e^{-a_{i}\left(v\right)}\right]}{a_{i}\left(v\right)}[\sum_{j=1}^{n}b_{ij}\left(v\right)f_{j}\left(0\right)\\ & & +\sum_{j=1}^{n}c_{ij}\left(v\right)g_{j}\left(0\right)+\overset{n}{\underset{j=1}{⋀}}\alpha_{ij}g_{j}\left(0\right)+\overset{n}{\underset{j=1}{⋀}}T_{ij}\mu_{j}\\ & & +\overset{n}{\underset{j=1}{⋁}}\beta_{ij}g_{j}\left(0\right)+\overset{n}{\underset{j=1}{⋁}}S_{ij}\mu_{j}+\sum_{j=1}^{n}d_{ij}\left(v\right)\sigma_{j}\left(0\right)\Delta w_{j}\left(v\right)+I_{i}\left(v\right)], \end{array}$$

where *i* = 1, 2, …, *n*, k∈Z. By Minkoswki inequality in Lemma 2, we have

$$\begin{array}{ccc} & & \|X^{0}{\|}_{p}\\ & \leq & \underset{1\leq i\leq n}{\text{max}}\underset{k\in Z}{\text{sup}}\{[E|\sum_{v=-\infty }^{k-1}\prod_{s=v+1}^{k-1}\frac{e^{-a_{i}\left(s\right)}\left[1-e^{-a_{i}\left(v\right)}\right]}{a_{i}\left(v\right)}\sum_{j=1}^{n}(b_{ij}\left(v\right)f_{j}\left(0\right)+c_{ij}\left(v\right)g_{j}\left(0\right)){|}^{p}{]}^{\frac{1}{p}}\\ & & +[E|\sum_{v=-\infty }^{k-1}\prod_{s=v+1}^{k-1}\frac{e^{-a_{i}\left(s\right)}\left[1-e^{-a_{i}\left(v\right)}\right]}{a_{i}\left(v\right)}(\overset{n}{\underset{j=1}{⋀}}\alpha_{ij}+\overset{n}{\underset{j=1}{⋁}}\beta_{ij})g_{j}\left(0\right){|}^{p}{]}^{\frac{1}{p}}\\ & & +[E|\sum_{v=-\infty }^{k-1}\prod_{s=v+1}^{k-1}\frac{e^{-a_{i}\left(s\right)}\left[1-e^{-a_{i}\left(v\right)}\right]}{a_{i}\left(v\right)}(\overset{n}{\underset{j=1}{⋀}}T_{ij}+\overset{n}{\underset{j=1}{⋁}}S_{ij})\mu_{j}{|}^{p}{]}^{\frac{1}{p}}\\ & & +[E|\sum_{v=-\infty }^{k-1}\prod_{s=v+1}^{k-1}\frac{e^{-a_{i}\left(s\right)}\left[1-e^{-a_{i}\left(v\right)}\right]}{a_{i}\left(v\right)}\sum_{j=1}^{n}d_{ij}\left(v\right)\sigma_{j}\left(0\right)\Delta w_{j}\left(v\right){|}^{p}{]}^{\frac{1}{p}}\\ & & +[E|\sum_{v=-\infty }^{k-1}\prod_{s=v+1}^{k-1}\frac{e^{-a_{i}\left(s\right)}\left[1-e^{-a_{i}\left(v\right)}\right]}{a_{i}\left(v\right)}I_{i}\left(v\right){|}^{p}{]}^{\frac{1}{p}}\}. \end{array}$$

From Lemma 6 and Hölder inequality in Lemma 3, it gets from the above inequality that

$$\begin{array}{ccc} \|X^{0}{\|}_{p} & \leq & \underset{1\leq i\leq n}{\text{max}}\underset{k\in Z}{\text{sup}}\{\frac{\left(1-e^{-\bar{a}}\right)}{\underline{a}\left(1-e^{-\underline{a}}\right)}[\sum_{j=1}^{n}({\bar{b}}_{ij}|f_{j}\left(0\right)|+{\bar{c}}_{ij}\left|g_{j}\left(0\right)\right|)\\ & & +\sum_{j=1}^{n}(|\alpha_{ij}\left|+\right|\beta_{ij}|)|g_{j}\left(0\right)|+\sum_{j=1}^{n}(\sum_{j=1}^{n}|T_{ij}\left|+\right|S_{ij}|)\left|\mu_{j}\right|+{\bar{I}}_{i}]\\ & & +\sum_{j=1}^{n}{\bar{d}}_{ij}\sigma_{j}\left(0\right)[\sum_{v=-\infty }^{k-1}\prod_{s=v+1}^{k-1}\frac{e^{-a_{i}\left(s\right)}\left[1-e^{-a_{i}\left(v\right)}\right]}{a_{i}\left(v\right)}{]}^{1-\frac{1}{p}}\\ & & \times [\sum_{v=-\infty }^{k-1}\prod_{s=v+1}^{k-1}\frac{e^{-a_{i}\left(s\right)}\left[1-e^{-a_{i}\left(v\right)}\right]}{a_{i}\left(v\right)}E{\left|\Delta w_{j}\left(v\right)\right|}^{p}{]}^{\frac{1}{p}}\}\\ & \leq & \frac{\left(1-e^{-\bar{a}}\right)}{\underline{a}\left(1-e^{-\underline{a}}\right)}\underset{1\leq i\leq n}{\text{max}}[\sum_{j=1}^{n}({\bar{b}}_{ij}|f_{j}\left(0\right)|+{\bar{c}}_{ij}\left|g_{j}\left(0\right)\right|)+\sum_{j=1}^{n}(|\alpha_{ij}\left|+\right|\beta_{ij}|)\left|g_{j}\left(0\right)\right|\\ & & +\sum_{j=1}^{n}(|T_{ij}\left|+\right|S_{ij}|)\left|\mu_{j}\right|+{\bar{I}}_{i}+\sum_{j=1}^{n}{\bar{d}}_{ij}\sigma_{j}\left(0\right)C_{p}^{\frac{1}{p}}]≔\alpha_{p}. \end{array}$$ (14)

It follows from (11), (12) and (13) that

$$\begin{array}{ccc} & & \|\Phi X-X^{0}{\|}_{p}\\ & \leq & \underset{1\leq i\leq n}{\text{max}}\underset{k\in Z}{\sup}\sum_{j=1}^{n}{\bar{b}}_{ij}L_{j}^{f}\{E[\sum_{v=-\infty }^{k-1}\prod_{s=v+1}^{k-1}\frac{e^{-a_{i}\left(s\right)}\left[1-e^{-a_{i}\left(v\right)}\right]}{a_{i}\left(v\right)}\left|x_{j}\left(v\right)\right|{]}^{p}{\}}^{\frac{1}{p}}\\ & & +\underset{1\leq i,j\leq n}{\text{max}}\underset{k\in Z}{\sup}\;D_{i}^{**}\{E[\sum_{v=-\infty }^{k-1}\prod_{s=v+1}^{k-1}\frac{e^{-a_{i}\left(s\right)}\left[1-e^{-a_{i}\left(v\right)}\right]}{a_{i}\left(v\right)}\left|x_{j}\left(v-\tau_{ij}\left(v\right)\right)\right|{]}^{p}{\}}^{\frac{1}{p}}\\ & & +\underset{1\leq i,j\leq n}{\text{max}}\underset{k\in Z}{\sup}\;K^{*}\{E[\sum_{v=-\infty }^{k-1}\prod_{s=v+1}^{k-1}\frac{e^{-a_{i}\left(s\right)}\left[1-e^{-a_{i}\left(v\right)}\right]}{a_{i}\left(v\right)}\left|x_{j}\left(v-\eta_{ij}\left(v\right)\right)\Delta w_{j}\left(v\right)\right|{]}^{p}{\}}^{\frac{1}{p}}, \end{array}$$

which yields from Lemma 3 that

$$\begin{array}{ccc} & & \|\Phi X-X^{0}{\|}_{p}\\ & \leq & \underset{1\leq i\leq n}{\text{max}}\underset{k\in Z}{\text{sup}}\sum_{j=1}^{n}{\bar{b}}_{ij}L_{j}^{f}\{[\sum_{v=-\infty }^{k-1}\prod_{s=v+1}^{k-1}\frac{e^{-a_{i}\left(s\right)}\left[1-e^{-a_{i}\left(v\right)}\right]}{a_{i}\left(v\right)}{]}^{p-1}\\ & & \times \sum_{v=-\infty }^{k-1}\prod_{s=v+1}^{k-1}\frac{e^{-a_{i}\left(s\right)}\left[1-e^{-a_{i}\left(v\right)}\right]}{a_{i}\left(v\right)}E|x_{j}\left(v\right){|}^{p}{\}}^{\frac{1}{p}}\\ & & +\underset{1\leq i,j\leq n}{\text{max}}\underset{k\in Z}{\text{sup}}D_{i}^{**}\{[\sum_{v=-\infty }^{k-1}\prod_{s=v+1}^{k-1}\frac{e^{-a_{i}\left(s\right)}\left[1-e^{-a_{i}\left(v\right)}\right]}{a_{i}\left(v\right)}{]}^{p-1}\\ & & \times \sum_{v=-\infty }^{k-1}\prod_{s=v+1}^{k-1}\frac{e^{-a_{i}\left(s\right)}\left[1-e^{-a_{i}\left(v\right)}\right]}{a_{i}\left(v\right)}E|x_{j}\left(v-\tau_{ij}\left(v\right)\right){|}^{p}{\}}^{\frac{1}{p}}\\ & & +\underset{1\leq i,j\leq n}{\text{max}}\underset{k\in Z}{\text{sup}}K^{*}\{[\sum_{v=-\infty }^{k-1}\prod_{s=v+1}^{k-1}\frac{e^{-a_{i}\left(s\right)}\left[1-e^{-a_{i}\left(v\right)}\right]}{a_{i}\left(v\right)}{]}^{p-1}\\ & & \times \sum_{v=-\infty }^{k-1}\prod_{s=v+1}^{k-1}\frac{e^{-a_{i}\left(s\right)}\left[1-e^{-a_{i}\left(v\right)}\right]}{a_{i}\left(v\right)}E|x_{j}\left(v-\eta_{ij}\left(v\right)\right)\Delta w_{j}\left(v\right){|}^{p}{\}}^{\frac{1}{p}}, \end{array}$$ (15)

where $D_{i}^{**}=D^{*}-\sum_{j=1}^{n}{\bar{b}}_{ij}L_{j}^{f}$, *i* = 1, 2, …, *n*. Applying Lemma 5 to the above inequality, it derives

$$\begin{array}{c} \|\Phi X-X^{0}{\|}_{p}\leq \frac{\left(1-e^{-\bar{a}}\right)}{\underline{a}\left(1-e^{-\underline{a}}\right)}\{D^{*}+K^{*}C_{p}^{\frac{1}{p}}\}{\left\|X\right\|}_{p}=r_{p}{\left\|X\right\|}_{p}\leq \frac{r_{p}\alpha_{p}}{1-r_{p}}. \end{array}$$ (16)

Hence, ∀*X* = {*x*_*i*_} ∈ Λ, it leads from (14) and (16) to

$$\begin{array}{c} {\left\|\Phi X\right\|}_{p}\leq \|X^{0}{\|}_{p}+{\left\|\Phi X-X^{0}\right\|}_{p}\leq \alpha_{p}+\frac{r_{p}\alpha_{p}}{1-r_{p}}=\frac{\alpha_{p}}{1-r_{p}}≔\beta_{p}. \end{array}$$ (17)

Similar to the argument as that in (17), it is easy to verify that BΛ is uniformly bounded and continuous. Together with the continuity of B, for any bounded sequence {*φ*_*n*_} in Λ, we know that there exists a subsequence $\left\{\varphi_{n_{k}}\right\}$ in Λ such that $\left\{B\left(\varphi_{n_{k}}\right)\right\}$ is convergent in B(Λ). Therefore, B is compact on Λ. Then condition (1) of Lemma 7 is satisfied.

The next step is proving condition (2) of Lemma 7. Now, we consist in proving the *p*-th mean almost periodicity of BX(·) and CX(·). Since *X*(⋅) is a *p*-th mean almost periodic sequence and all coefficients in system (5) are almost periodic sequences, for any *ϵ* > 0 there exists *l*_*ϵ*_ > 0 such that every interval of length *l*_*ϵ*_ > 0 contains a *ω* with the property that

$$\begin{array}{c} {}[E|x_{i}\left(k+\omega \right)-x_{i}{\left(k\right)|}^{p}{]}^{\frac{1}{p}}<\epsilon ,\;\left|a_{i}\left(k+\omega \right)-a_{i}\left(k\right)\right|<\epsilon , \end{array}$$

$$\begin{array}{c} |b_{ij}\left(k+\omega \right)-b_{ij}\left(k\right)|<\epsilon ,\;|c_{ij}\left(k+\omega \right)-c_{ij}\left(k\right)|<\epsilon ,\;|d_{ij}\left(k+\omega \right)-d_{ij}\left(k\right)|<\epsilon , \end{array}$$

$$\begin{array}{c} |\tau_{ij}\left(k+\omega \right)-\tau_{ij}\left(k\right)|<\epsilon ,\;|\eta_{ij}\left(k+\omega \right)-\eta_{ij}\left(k\right)|<\epsilon ,\;|I_{i}\left(k+\omega \right)-I_{i}\left(k\right)|<\epsilon , \end{array}$$

where *i*, *j* = 1, 2, …, *n*, k∈Z. By (12), (13) and (*H*_2_), we could easily find a positive constant *M* such that

$$\begin{array}{ccc} {}[{E|\left(BX\right)}_{i}\left(k+\omega \right)-{\left(BX\right)}_{i}{\left(k\right)|}^{p}{]}^{\frac{1}{p}} & \leq & M\underset{1\leq i\leq n}{\text{max}}\underset{k\in Z}{\text{sup}}\;[E|x_{i}\left(k+\omega \right)-x_{i}{\left(k\right)|}^{p}{]}^{\frac{1}{p}}<M\epsilon ,\; \end{array}$$ (18)

$$\begin{array}{ccc} {}[{E|\left(CX\right)}_{i}\left(k+\omega \right)-{\left(CX\right)}_{i}{\left(k\right)|}^{p}{]}^{\frac{1}{p}} & \leq & M\underset{1\leq i\leq n}{\text{max}}\underset{k\in Z}{\text{sup}}\;[E|x_{i}\left(k+\omega \right)-x_{i}{\left(k\right)|}^{p}{]}^{\frac{1}{p}}<M\epsilon ,\; \end{array}$$ (19)

where *i* = 1, 2, …, *n*, k∈Z. From (18) and (19), BX(·) and CX(·) are *p*-th mean almost periodic processes. Further, by (17), it is easy to obtain that BX+CY∈Λ, ∀*X*, *Y* ∈ Λ. Then condition (2) of Lemma 7 holds.

Finally, ∀*X* = {*x*_*i*_}, *Y* = {*y*_*i*_} ∈ Λ, from (13), it yields

$$\begin{array}{ccc} {\left\|CX-CY\right\|}_{p} & \leq & \frac{\left[1-e^{-\bar{a}}\right]}{\underline{a}}\underset{1\leq i\leq n}{\text{max}}\underset{k\in Z}{\text{sup}}\{E[\sum_{v=-\infty }^{k-1}\prod_{s=v+1}^{k-1}e^{-\underline{a}}\\ & & \times \sum_{j=1}^{n}d_{ij}\left(v\right)(\sigma_{j}\left(x_{j}\left(v-\eta_{ij}\left(v\right)\right)\right)-\sigma_{j}\left(y_{j}\left(v-\eta_{ij}\left(v\right)\right)\right))\Delta w_{j}\left(v\right){]}^{p}{\}}^{\frac{1}{p}}\\ & \leq & \frac{\left[1-e^{-\bar{a}}\right]}{\underline{a}}\underset{1\leq i,j\leq n}{\text{max}}\underset{k\in Z}{\text{sup}}\;K^{*}\{[\sum_{v=-\infty }^{k-1}\prod_{s=v+1}^{k-1}e^{-\underline{a}}{]}^{p-1}\\ & & \times \sum_{v=-\infty }^{k-1}\prod_{s=v+1}^{k-1}e^{-\underline{a}}E|\left[x_{j}\left(v-\eta_{ij}\left(v\right)\right)-y_{j}\left(v-\eta_{ij}\left(v\right)\right)\right]\Delta w_{j}\left(v\right){|}^{p}{\}}^{\frac{1}{p}}\\ & \leq & \frac{K^{*}C_{p}^{\frac{1}{p}}\left(1-e^{-\bar{a}}\right)}{\underline{a}\left(1-e^{-\underline{a}}\right)}{\left\|X-Y\right\|}_{p}\\ & \leq & r_{p}{\left\|X-Y\right\|}_{p}. \end{array}$$ (20)

In view of (*H*_3_), C is a contraction mapping. Hence condition (3) of Lemma 7 is satisfied. Therefore, all conditions in Lemma 7 hold. By Lemma 7, system (5) has a *p*-th mean almost periodic sequence solution. This completes the proof.

## *p*-th moment global exponential stability

Suppose that *X* = {*x*_*i*_} with initial value *φ* = {*φ*_*i*_} and $X^{*}=\left\{x_{i}^{*}\right\}$ with initial value $\varphi^{*}=\left\{\varphi_{i}^{*}\right\}$ are arbitrary two solutions of system (5). For convenience, let $\gamma_{p}={\text{max}}_{1\leq i\leq n}\;{\text{sup}}_{s\in {\left[-\mu_{0},0\right]}_{\mathbb{Z}}}\{{\left(E|\varphi_{i}(s)-\varphi_{i}^{*}(s){|}^{p}\right)}^{\frac{1}{p}}\}$, $\mu_{0}={\text{max}}_{1\leq i,j\leq n}\left\{{\bar{\tau }}_{ij},{\bar{\eta }}_{ij}\right\}$.

**Definition 2**. ([9]) System (5) is said to be *p*-th moment global exponential stability if there are positive constants *k*_0_, *M* and λ such that

$$\begin{array}{c} |X\left(k\right)-X^{*}{\left(k\right)|}_{p}=\underset{1\leq i\leq n}{\text{max}}(E|x_{i}\left(k\right)-x_{i}^{*}{\left(k\right)|}^{p}{)}^{\frac{1}{p}}<M\gamma_{p}e^{-\lambda k},\;\forall k>k_{0},\;\;k\in Z. \end{array}$$

The 2-nd moment global exponential stability will be called square-mean global exponential stability.

**Theorem 2**. *Assume that* (*H*_1_)-(*H*_3_) *hold, then system* (5) *is p-th moment globally exponentially stable, p* > 1.

*Proof*. By Lemma 1, it follows that

$$\begin{array}{ccc} & & |x_{i}\left(k\right)-x_{i}^{*}\left(k\right)|\\ & \leq & \prod_{s=0}^{k-1}e^{-a_{i}\left(s\right)}|\varphi_{i}\left(0\right)-\varphi_{i}^{*}\left(0\right)|+\frac{\left(1-e^{-\bar{a}}\right)}{\underline{a}}\sum_{v=0}^{k-1}\prod_{s=v+1}^{k-1}e^{-a_{i}\left(s\right)}\sum_{j=1}^{n}\{{\bar{b}}_{ij}L_{j}^{f}\left|x_{j}\left(v\right)-x_{j}^{*}\left(v\right)\right|\\ & & +({\bar{c}}_{ij}+|\alpha_{ij}\left|+\right|\beta_{ij}\left|\right)L_{j}^{g}\left|x_{j}\left(v-\tau_{ij}\left(v\right)\right)-x_{j}^{*}\left(v-\tau_{ij}\left(v\right)\right)\right|\\ & & +{\bar{d}}_{ij}L_{j}^{\sigma }|x_{j}\left(v-\eta_{ij}\left(v\right)\right)-x_{j}^{*}\left(v-\eta_{ij}\left(v\right)\right)||\Delta w_{j}\left(v\right)|\}\\ & \leq & e^{-\underline{a}k}|\varphi_{i}\left(0\right)-\varphi_{i}^{*}\left(0\right)|+\frac{\left(1-e^{-\bar{a}}\right)}{\underline{a}}\sum_{v=0}^{k-1}e^{-\underline{a}\left(k-v-1\right)}\sum_{j=1}^{n}\{{\bar{b}}_{ij}L_{j}^{f}\left|x_{j}\left(v\right)-x_{j}^{*}\left(v\right)\right|\\ & & +({\bar{c}}_{ij}+|\alpha_{ij}\left|+\right|\beta_{ij}\left|\right)L_{j}^{g}\left|x_{j}\left(v-\tau_{ij}\left(v\right)\right)-x_{j}^{*}\left(v-\tau_{ij}\left(v\right)\right)\right|\\ & & +{\bar{d}}_{ij}L_{j}^{\sigma }|x_{j}\left(v-\eta_{ij}\left(v\right)\right)-x_{j}^{*}\left(v-\eta_{ij}\left(v\right)\right)||\Delta w_{j}\left(v\right)|\}, \end{array}$$ (21)

where *i* = 1, 2, …, *n*, $k\in {\left[1,+\infty \right)}_{Z}$. For convenience, let $a_{0}=\frac{1-e^{-\bar{a}}}{\underline{a}}$ and *Z*(*k*) = {*z*_*i*_(*k*)}, $z_{i}\left(k\right)=x_{i}\left(k\right)-x_{i}^{*}\left(k\right)$, *i* = 1, 2, …, *n*, k∈Z. By Lemmas 2 and 3, it gets from (21) that

$$\begin{array}{ccc} {\left|Z\left(k\right)\right|}_{p} & = & |X\left(k\right)-X^{*}{\left(k\right)|}_{p}\\ & \leq & e^{-\underline{a}k}\gamma_{p}\\ & & +\underset{1\leq i\leq n}{\text{max}}\sum_{j=1}^{n}a_{0}{\bar{b}}_{ij}L_{j}^{f}\{[\sum_{s=0}^{k-1}e^{-\underline{a}\left(k-s-1\right)}{]}^{p-1}\\ & & \sum_{s=0}^{k-1}e^{-\underline{a}\left(k-s-1\right)}E|x_{j}\left(s\right)-x_{j}^{*}\left(s\right){|}^{p}{\}}^{\frac{1}{p}}\\ & & +\underset{1\leq i\leq n}{\text{max}}\sum_{j=1}^{n}a_{0}({\bar{c}}_{ij}+|\alpha_{ij}\left|+\right|\beta_{ij}\left|\right)L_{j}^{g}\{[\sum_{s=0}^{k-1}e^{-\underline{a}\left(k-s-1\right)}{]}^{p-1}\\ & & \times \sum_{s=0}^{k-1}e^{-\underline{a}\left(k-s-1\right)}E|x_{j}\left(s-\tau_{ij}\left(s\right)\right)-x_{j}^{*}\left(s-\tau_{ij}\left(s\right)\right){|}^{p}{\}}^{\frac{1}{p}}\\ & & +\underset{1\leq i\leq n}{\text{max}}\sum_{j=1}^{n}a_{0}{\bar{d}}_{ij}L_{j}^{\sigma }\{[\sum_{s=0}^{k-1}e^{-\underline{a}\left(k-s-1\right)}{]}^{p-1}\\ & & \times \sum_{s=0}^{k-1}e^{-\underline{a}\left(k-s-1\right)}E|\left[x_{j}\left(s-\eta_{ij}\left(s\right)\right)-x_{j}^{*}\left(s-\eta_{ij}\left(s\right)\right)\right]\Delta w_{j}\left(s\right){|}^{p}{\}}^{\frac{1}{p}}\\ & \leq & e^{-\underline{a}k}\gamma_{p}+\underset{1\leq i\leq n}{\text{max}}\sum_{j=1}^{n}a_{0}{\bar{b}}_{ij}L_{j}^{f}\{[\sum_{s=0}^{k-1}e^{-\underline{a}\left(k-s-1\right)}{]}^{p-1}\sum_{s=0}^{k-1}e^{-\underline{a}\left(k-s-1\right)}{\left|Z\left(s\right)\right|}_{p}^{p}{\}}^{\frac{1}{p}}\\ & & +\underset{1\leq i\leq n}{\text{max}}\sum_{j=1}^{n}a_{0}({\bar{c}}_{ij}+|\alpha_{ij}\left|+\right|\beta_{ij}\left|\right)L_{j}^{g}\{[\sum_{s=0}^{k-1}e^{-\underline{a}\left(k-s-1\right)}{]}^{p-1}\\ & & \sum_{s=0}^{k-1}e^{-\underline{a}\left(k-s-1\right)}{\left|Z\left(s-\tau_{ij}\left(s\right)\right)\right|}_{p}^{p}{\}}^{\frac{1}{p}}\\ & & +\underset{1\leq i\leq n}{\text{max}}\sum_{j=1}^{n}a_{0}C_{p}^{\frac{1}{p}}{\bar{d}}_{ij}L_{j}^{\sigma }\{[\sum_{s=0}^{k-1}e^{-\underline{a}\left(k-s-1\right)}{]}^{p-1}\\ & & \sum_{s=0}^{k-1}e^{-\underline{a}\left(k-s-1\right)}{\left|Z\left(s-\eta_{ij}\left(s\right)\right)\right|}_{p}^{p}{\}}^{\frac{1}{p}}. \end{array}$$ (22)

Be aware of (*H*_3_) in Theorem 1, there exists a constant λ > 0 small enough such that

$$\begin{array}{c} \underset{1\leq i\leq n}{\text{max}}\sum_{j=1}^{n}\frac{e^{\lambda }a_{0}}{1-e^{-(\underline{a}-2p\lambda )}}[{\bar{b}}_{ij}L_{j}^{f}+e^{\mu_{0}\lambda }({\bar{c}}_{ij}+|\alpha_{ij}\left|+\right|\beta_{ij}\left|\right)L_{j}^{g}+e^{\mu_{0}\lambda }C_{p}^{\frac{1}{p}}{\bar{d}}_{ij}L_{j}^{\sigma }]\overset{\text{def}}{=}\rho \leq 1. \end{array}$$

Next, we claim that there exists a constant *M*_0_ > 1 such that

$$\begin{array}{c} {\left|Z\left(k\right)\right|}_{p}\leq M_{0}\gamma_{p}e^{-\lambda k},\;\forall k\in {\left[-\mu_{0},+\infty \right)}_{Z}. \end{array}$$ (23)

If (23) is invalid, then there must exist $k_{0}\in {\left(0,+\infty \right)}_{Z}$ such that

$$\begin{array}{c} |Z\left(k_{0}\right){|}_{p}>M_{0}\gamma_{p}e^{-\lambda k_{0}} \end{array}$$ (24)

and

$$\begin{array}{c} {\left|Z\left(k\right)\right|}_{p}\leq M_{0}\gamma_{p}e^{-\lambda k},\;\forall k\in {\left[-\mu_{0},k_{0}\right)}_{Z}. \end{array}$$ (25)

In view of (22), it follows from (25) that

$$\begin{array}{ccc} |Z\left(k_{0}\right){|}_{p} & \leq & e^{-\underline{a}k_{0}}\gamma_{p}\\ & & +\underset{1\leq i\leq n}{\text{max}}\sum_{j=1}^{n}a_{0}{\bar{b}}_{ij}L_{j}^{f}M_{0}\gamma_{p}\{[\sum_{s=0}^{k_{0}-1}e^{-\underline{a}\left(k_{0}-s-1\right)}{]}^{p-1}\sum_{s=0}^{k_{0}-1}e^{-\underline{a}\left(k_{0}-s-1\right)}e^{-p\lambda s}{\}}^{\frac{1}{p}}\\ & & +\underset{1\leq i\leq n}{\text{max}}\sum_{j=1}^{n}a_{0}M_{0}\gamma_{p}[({\bar{c}}_{ij}+|\alpha_{ij}\left|+\right|\beta_{ij}\left|\right)L_{j}^{g}+C_{p}^{\frac{1}{p}}{\bar{d}}_{ij}L_{j}^{\sigma }]\\ & & \times \{[\sum_{s=0}^{k_{0}-1}e^{-\underline{a}\left(k_{0}-s-1\right)}{]}^{p-1}\sum_{s=0}^{k_{0}-1}e^{-\underline{a}\left(k_{0}-s-1\right)}e^{-p\lambda (s-\mu_{0})}{\}}^{\frac{1}{p}}\\ & \leq & e^{-\underline{a}k_{0}}\gamma_{p}\\ & & +\underset{1\leq i\leq n}{\text{max}}\sum_{j=1}^{n}a_{0}{\bar{b}}_{ij}L_{j}^{f}M_{0}\gamma_{p}e^{-\lambda k_{0}}e^{\lambda }[\frac{1-e^{-\underline{a}k_{0}}}{1-e^{-\underline{a}}}{]}^{1-\frac{1}{p}}[\sum_{s=0}^{k_{0}-1}e^{-\left(\underline{a}-p\lambda \right)\left(k_{0}-s-1\right)}{]}^{\frac{1}{p}}\\ & & +\underset{1\leq i\leq n}{\text{max}}\sum_{j=1}^{n}a_{0}M_{0}\gamma_{p}[({\bar{c}}_{ij}+|\alpha_{ij}\left|+\right|\beta_{ij}\left|\right)L_{j}^{g}+C_{p}^{\frac{1}{p}}{\bar{d}}_{ij}L_{j}^{\sigma }]\\ & & \times e^{-\lambda k_{0}}e^{(\mu_{0}+1)\lambda }[\frac{1-e^{-\underline{a}k_{0}}}{1-e^{-\underline{a}}}{]}^{1-\frac{1}{p}}[\sum_{s=0}^{k_{0}-1}e^{-\left(\underline{a}-p\lambda \right)\left(k_{0}-s-1\right)}{]}^{\frac{1}{p}}\\ & \leq & e^{-\underline{a}k_{0}}\gamma_{p}+\underset{1\leq i\leq n}{\text{max}}\sum_{j=1}^{n}a_{0}M_{0}\gamma_{p}e^{-\lambda k_{0}}[{\bar{b}}_{ij}L_{j}^{f}+e^{\mu_{0}\lambda }({\bar{c}}_{ij}+|\alpha_{ij}\left|+\right|\beta_{ij}\left|\right)L_{j}^{g}\\ & & +e^{\mu_{0}\lambda }C_{p}^{\frac{1}{p}}{\bar{d}}_{ij}L_{j}^{\sigma }]e^{\lambda }[\frac{1-e^{-\underline{a}k_{0}}}{1-e^{-\underline{a}}}{]}^{1-\frac{1}{p}}[\frac{1-e^{-\left(\underline{a}-p\lambda \right)k_{0}}}{1-e^{-(\underline{a}-p\lambda )}}{]}^{\frac{1}{p}}\\ & \leq & M_{0}\gamma_{p}e^{-\lambda k_{0}}\{\frac{1}{M_{0}}e^{-\left(\underline{a}-\lambda \right)k_{0}}+\underset{1\leq i\leq n}{\text{max}}\sum_{j=1}^{n}a_{0}[{\bar{b}}_{ij}L_{j}^{f}\\ & & +e^{\mu_{0}\lambda }({\bar{c}}_{ij}+|\alpha_{ij}\left|+\right|\beta_{ij}\left|\right)L_{j}^{g}+e^{\mu_{0}\lambda }C_{p}^{\frac{1}{p}}{\bar{d}}_{ij}L_{j}^{\sigma }]\frac{e^{\lambda }\left[1-e^{-\left(\underline{a}-\lambda \right)k_{0}}\right]}{1-e^{-(\underline{a}-p\lambda )}}\\ & \leq & M_{0}\gamma_{p}e^{-\lambda k_{0}}\{e^{-\left(\underline{a}-\lambda \right)k_{0}}+\rho \left[1-e^{-\left(\underline{a}-\lambda \right)k_{0}}\right]\}\\ & \leq & M_{0}\gamma_{p}e^{-\lambda k_{0}}. \end{array}$$ (26)

In the fourth inequality from the bottom of (26), we use the fact ${\left[1-e^{-\underline{a}k_{0}}\right]}^{1-\frac{1}{p}}{\left[1-e^{-\left(\underline{a}-p\lambda \right)k_{0}}\right]}^{\frac{1}{p}}\leq 1-e^{-\left(\underline{a}-\lambda \right)k_{0}}$ and ${\left[1-e^{-\underline{a}}\right]}^{\frac{1}{p}}\geq {\left[1-e^{-(\underline{a}-p\lambda )}\right]}^{\frac{1}{p}}$. (26) contradicts (24). Hence, (23) is satisfied. Therefore, system (5) is *p*-th moment globally exponentially stable. This completes the proof.

Together with Theorem 1, we have

**Theorem 3**. *Assume that all conditions in Theorem* 1 *hold, then system* (5) *admits a p-th mean almost periodic sequence solution, which is p-th moment globally exponentially stable. Further, if all coefficients in system* (5) *are periodic sequences, then system* (5) *admits at least one p-th mean periodic sequence solution, which is globally exponentially stable*.

*Proof*. The result can be easily obtained by Theorem 2, so we omit it. This completes the proof.

In system (5), if we remove the effects of uncertain factors, then the following deterministic model is obtained:

$$\begin{array}{ccc} x_{i}\left(k+1\right) & = & e^{-a_{i}\left(k\right)}x_{i}\left(k\right)+\frac{1-e^{-a_{i}\left(k\right)}}{a_{i}\left(k\right)}[\sum_{j=1}^{n}b_{ij}\left(k\right)f_{j}\left(x_{j}\left(k\right)\right)\\ & & +\sum_{j=1}^{n}c_{ij}\left(k\right)g_{j}\left(x_{j}\left(k-\tau_{ij}\left(k\right)\right)\right)+I_{i}\left(k\right)], \end{array}$$ (27)

where k∈Z, *i* = 1, 2, …, *n*.

Define

$$\begin{array}{c} \hat{r}≔\underset{1\leq i\leq n}{\text{max}}\frac{\left(1-e^{-{\bar{a}}_{i}}\right)}{{\underline{a}}_{i}\left(1-e^{-{\underline{a}}_{i}}\right)}\sum_{j=1}^{n}\left({\bar{b}}_{ij}L_{j}^{f}+{\bar{c}}_{ij}L_{j}^{g}\right). \end{array}$$

**Corollary 1**. *Assume that* (*H*_1_) *and* (7) and (8) *in* (*H*_2_) *hold. Suppose further that all of coefficients of model* (27) *are almost periodic sequences, and* $\hat{r}<1$, *then model* (27) *admits at least one almost periodic sequence solution, which is globally exponentially stable. Moreover, if all of coefficients of model* (27) *are periodic sequences, then model* (27) *admits at least one periodic solution, which is globally exponentially stable*.

**Remark 1**. In literature [52], Huang et al. studied model (27) with *c*_*ij*_ ≡ 0(*i*, *j* = 1, 2, …, *n*) and obtained some sufficient conditions for the existence of a unique almost periodic sequence solution which is globally attractive. In [53], they considered system (4) and studied the dynamics of 2^*N*^ almost periodic sequence solutions. But neither of them considered the uncertain factors. Therefore, the work in this paper complements the corresponding results in [52, 53].

**Remark 2**. Assume that *X*(*k*) = (*x*_1_(*k*), *x*_2_(*k*), …, *x*_*n*_(*k*)) is a solution of (27), the length of *X*(*k*) is usually measured by ${\left\|X\right\|}_{\infty }={\text{sup}}_{k\in R}{\text{max}}_{1\leq i\leq n}\;\left|x_{i}\left(k\right)\right|$. However, if *X*(*k*) is a solution of stochastic system (5), its length should not be measured by ‖*X*‖_∞_ because *X*(*k*) is a random variable. In this paper, we use norm ${\left\|X\right\|}_{p}={\text{max}}_{1\leq i\leq n}{\text{sup}}_{k\in Z}\left(E\right|x_{i}\left(k\right){{|}^{p})}^{\frac{1}{p}}\;\left(p>1\right)$ for random variable *X*(*k*). Owing to the expectation *E* and order *p* in ‖*X*‖_*p*_, the computing processes of this paper are more complicated than that in literatures [49–55]. It is worth mentioning that Minkoswki inequality in Lemma 2 and Hölder inequality in Lemma 3 are crucial to the computing processes. The facts above are obvious from the computations of (14), (15), (22) and (26) in Theorems 1 and 2. Further, the stochastic term *d*_*ij*_*σ*_*j*_Δ*w*_*j*_(*i*, *j* = 1, 2, …, *n*) in system (5) also increases the complexity of computing. This point is also clear from the computations of (20) and (22) in Theorems 1 and 2.

## Stochastic stabilization

In this section, we consider the following stochastic cellular neural networks:

$$dx_{i}\left(t\right)=[-a_{i}\left(t\right)x_{i}\left(t\right)+\sum_{j=1}^{n}b_{ij}\left(t\right)f_{j}\left(x_{j}\left(t\right)\right)+I_{i}\left(t\right)]dt+\kappa x_{i}\left(t\right)dw\left(t\right),$$ (28)

where *w*(*t*) is a standard Brownian motion, t∈R, *i* = 1, 2, …, *n*.

Let *κ* = 0 in system (28), the following deterministic cellular neural networks is derived:

$$\frac{dx_{i}\left(t\right)}{dt}=-a_{i}\left(t\right)x_{i}\left(t\right)+\sum_{j=1}^{n}b_{ij}\left(t\right)f_{j}\left(x_{j}\left(t\right)\right)+I_{i}\left(t\right),$$ (29)

where t∈R, *i* = 1, 2, …, *n*. Noting that the unique distinction between (28) and (29) is the stochastic disturbance.

### The semi-discretization models of systems (28) and (29)

Regarding the following stochastic differential equations (SDEs):

du(t)=-a(t)u(t)dt+F(t,u(t))dt+κu(t)dw(t),t∈R,

which yields the following SDEs with piecewise constant arguments:

du(t)=-a([t])u(t)dt+F([t],u([t]))dt+κu(t)dw(t),

where t∈R, [*t*] denotes the integer part of *t*. For each t∈R, there exists an integer k∈Z such that *k* ≤ *t* < *k* + 1. Then the above equation becomes

du(t)=-a(k)u(t)dt+F(k,u(k))dt+κu(t)dw(t),t∈R,k∈Z. (30)

Let *z*_*k*_(*t*) = *a*(*k*)*t* + 0.5*κ*^2^*t* − *κw*(*t*), ∀t∈R, k∈Z. By using Itô formula and formula of integration by parts in stochastic theory, it obtains from (30) that

$$\begin{array}{ccc} d(e^{z_{k}\left(t\right)}u\left(t\right)) & = & u\left(t\right)de^{z_{k}\left(t\right)}+e^{z_{k}\left(t\right)}du\left(t\right)+\left(de^{z_{k}\left(t\right)}\right)\cdot \left(du\left(t\right)\right)\\ & = & \left(a\left(k\right)+0.5\kappa^{2}\right)u\left(t\right)e^{z_{k}\left(t\right)}dt-\kappa u\left(t\right)e^{z_{k}\left(t\right)}dw\left(t\right)\\ & & +0.5\kappa^{2}u\left(t\right)e^{z_{k}\left(t\right)}dt+e^{z_{k}\left(t\right)}du\left(t\right)-\kappa^{2}u\left(t\right)e^{z_{k}\left(t\right)}dt\\ & = & e^{z_{k}\left(t\right)}F\left(k,u\left(k\right)\right)dt,\;t\in R,k\in Z. \end{array}$$

Integrating the above equation from *k* to *t* and letting *t* → *k* + 1, the following equation is obtained:

$$\begin{array}{ccc} u(k+1) & = & e^{p(k)}u\left(k\right)+e^{-z_{k}\left(k+1\right)}F\left(k,u\left(k\right)\right)\int_{k}^{k+1}e^{z_{k}\left(s\right)}ds\\ & \approx & e^{p(k)}u\left(k\right)+\frac{\left(1-e^{-a\left(k\right)-0.5\kappa^{2}}\right)e^{\kappa \Delta w(k)}}{a\left(k\right)+0.5\kappa^{2}}F\left(k,u\left(k\right)\right), \end{array}$$ (31)

where *p*(*k*) = −*a*(*k*) − 0.5*κ*^2^ + *κ*Δ*w*(*k*), Δ*w*(*k*) = *w*(*k* + 1) − *w*(*k*), k∈Z. In (31), we use the fact $\int_{k}^{k+1}e^{z_{k}\left(s\right)}ds\approx e^{-\kappa w(k)}\int_{k}^{k+1}e^{a\left(k\right)s+0.5\kappa^{2}s}ds$, k∈Z.

By a similar discussion as that in system (31), we gets the semi-discrete analogue for system (28) as follows:

$$\begin{array}{cc} x_{i}\left(k+1\right) & =e^{p_{i}\left(k\right)}x_{i}\left(k\right)\\ & \;+\frac{\left(1-e^{-a_{i}\left(k\right)-0.5\kappa^{2}}\right)e^{\kappa \Delta w(k)}}{a_{i}\left(k\right)+0.5\kappa^{2}}[\sum_{j=1}^{n}b_{ij}\left(t\right)f_{j}\left(x_{j}\left(t\right)\right)+I_{i}\left(k\right)], \end{array}$$ (SM)

where *p*_*i*_(*k*) = −*a*_*i*_(*k*) − 0.5*κ*^2^ + *κ*Δ*w*(*k*), Δ*w*(*k*) = *w*(*k* + 1) − *w*(*k*), k∈Z, *i* = 1, 2, …, *n*.

Let *κ* = 0 in system (SM), the semi-discrete analogue for system (29) is obtained as follows:

$$\begin{array}{c} x_{i}\left(k+1\right)=e^{-a_{i}\left(k\right)}x_{i}\left(k\right)+\frac{1-e^{-a_{i}\left(k\right)}}{a_{i}\left(k\right)}[\sum_{j=1}^{n}b_{ij}\left(t\right)f_{j}\left(x_{j}\left(t\right)\right)+I_{i}\left(k\right)], \end{array}$$ (DM)

where k∈Z, *i* = 1, 2, …, *n*. Also, the unique difference between (SM) and (DM) is the stochastic disturbance.

### Stability analysis of systems (SM) and (DM)

Assume that *X* = {*x*_*i*_} with initial value $X_{0}=\left\{x_{i0}\right\}\in R^{n}$ and $X^{*}=\left\{x_{i}^{*}\right\}$ with initial value $X_{0}^{*}=\left\{x_{i0}^{*}\right\}\in R^{n}$ are arbitrary two solutions of system (SM) or (DM).

**Definition 3**. ([9]) System (SM) or (DM) is said to be exponential stability if

$$\begin{array}{c} \lim_{k\to +\infty }\frac{\ln[\sum_{i=1}^{n}\left|x_{i}\left(k\right)-x_{i}^{*}\left(k\right)\right|]}{k}<0,\;\forall X_{0},X_{0}^{*}\in R^{n}. \end{array}$$

System (SM) or (DM) is said to be exponential instability if

$$\begin{array}{c} \lim_{k\to +\infty }\frac{\ln[\sum_{i=1}^{n}\left|x_{i}\left(k\right)-x_{i}^{*}\left(k\right)\right|]}{k}>0,\;\forall X_{0},X_{0}^{*},X_{0}-X_{0}^{*}\in R^{n}\backslash \left\{0\right\}. \end{array}$$

**Lemma 8**. ([9]) Assume that *w* is a standard Brownian motion, then *w*(0) = 0 and $\lim_{t\to \infty }\frac{w(t)}{t}=0$, a.s‥

**Theorem 4**. *Assume that* (*H*_2_) *holds. Suppose further that*

(*H*_4_) $\Theta ={\text{max}}_{1\leq i\leq n}[e^{-a_{i}^{-}-0.5\kappa^{2}}+\frac{1}{{\underline{a}}_{i}+0.5\kappa^{2}}\sum_{j=1}^{n}{\bar{b}}_{ij}L_{j}^{f}]<1$, *where* $a_{i}^{-}=\min_{k\in Z}a_{i}\left(k\right)$, *i* = 1, 2, …, *n*.

*Then system* (SM) *is exponentially stable*.

*Proof*. From (SM), it gets

$$\begin{array}{ccc} & & |x_{i}\left(k+1\right)-x_{i}^{*}\left(k+1\right)|\\ & \leq & e^{p_{i}\left(k\right)}|x_{i}\left(k\right)-x_{i}^{*}\left(k\right)|+\frac{\left(1-e^{-a_{i}\left(k\right)-0.5\kappa^{2}}\right)e^{\kappa \Delta w(k)}}{a_{i}\left(k\right)+0.5\kappa^{2}}\sum_{j=1}^{n}{\bar{b}}_{ij}L_{j}^{f}\left|x_{j}\left(k\right)-x_{j}^{*}\left(k\right)\right|\\ & \leq & \Theta e^{\kappa \Delta w(k)}\underset{1\leq i\leq n}{\text{max}}\left|x_{i}\left(k\right)-x_{i}^{*}\left(k\right)\right|,\;i=1,2,\ldots ,n, \end{array}$$

which derives

$$\begin{array}{ccc} \underset{1\leq i\leq n}{\text{max}}\left|x_{i}\left(k\right)-x_{i}^{*}\left(k\right)\right| & \leq & \Theta^{k}e^{\kappa w(k)}\underset{1\leq i\leq n}{\text{max}}\left|x_{i}\left(0\right)-x_{i}^{*}\left(0\right)\right|,\;k\in Z, \end{array}$$

which implies

$$\begin{array}{ccc} \frac{\text{ln}\;[{\text{max}}_{1\leq i\leq n}\left|x_{i}\left(k\right)-x_{i}^{*}\left(k\right)\right|]}{k} & \leq & \text{ln}\;\Theta +\frac{\left|\kappa w(k)\right|}{k}+\frac{\text{ln}\;\gamma_{0}}{k},\;k\in {\left[1,+\infty \right)}_{Z}. \end{array}$$

From Lemma 8, it leads to

$$\begin{array}{ccc} \lim_{k\to +\infty }\frac{\text{ln}\;[{\text{max}}_{1\leq i\leq n}\left|x_{i}\left(k\right)-x_{i}^{*}\left(k\right)\right|]}{k} & \leq & \text{ln}\;\Theta <0. \end{array}$$

Then system (SM) is exponential stability. This completes the proof.

Let *κ* = 0 in Theorem 4, it has

**Theorem 5**. *Assume that* (*H*_2_) *holds. Suppose further that*

(*H*_5_) ${\text{max}}_{1\leq i\leq n}[e^{-a_{i}^{-}}+\frac{1}{{\underline{a}}_{i}}\sum_{j=1}^{n}{\bar{b}}_{ij}L_{j}^{f}]<1$.

*Then system* (DM) *is exponentially stable*.

Similar to the argument as that in Theorem 4, the exponential instability of system (DM) is easily derived as follows:

**Theorem 6**. *Assume that* (*H*_2_) *holds. Suppose further that*

(*H*_6_) $\min_{1\leq i\leq n}[e^{-a_{i}^{+}}-\frac{1}{{\underline{a}}_{i}}\sum_{j=1}^{n}{\bar{b}}_{ij}L_{j}^{f}]>1$, where $a_{i}^{+}={\text{max}}_{k\in Z}\;a_{i}\left(k\right)$, *i* = 1, 2, …, *n*.

*Then system* (DM) *is exponentially instable*.

**Definition 4**. ([9]) Assume that system (DM) is exponential instability and there exists a suitable stochastic disturbance coefficient *κ* ensuring that system (SM) is exponential stable, then system (SM) is a stochastic stabilization system of system (DM).

Together with Theorems 4 and 6, it gains

**Theorem 7**. *Assume that* (*H*_2_), (*H*_4_) *and* (*H*_6_) *are satisfied. Then system* (SM) *is a stochastic stabilization system of system* (DM).

**Remark 3**. If (*H*_6_) is valid, (DM) is exponentially instable. Meanwhile, (*H*_5_) is invalid. By viewing (*H*_4_), one could select a suitable stochastic disturbance coefficient *κ* ensuring that (*H*_4_) is satisfied, which yields system (SM) is exponentially stable. Therefore, stochastic disturbance could be a useful method, which brings unstable system to be stable. More details could be observed in Example 2.

## Examples and computer simulations

**Example 1**. Consider the following continuous-time uncertain cellular neural networks with random perturbations and fuzzy operations:

$$\begin{array}{c} \left\{ \begin{array}{ccc} dx_{1}\left(t\right) & = & [-x_{1}\left(t\right)+0.01\;\text{sin}\;\left(\sqrt{5}t\right)\;\text{sin}\;\left(x_{1}\left(t\right)\right)+0.05\;\text{sin}\;\left(\sqrt{7}t\right)\;\text{cos}\;\left(x_{2}\left(t-1\right)\right)\\ & & +\overset{2}{\underset{j=1}{⋀}}0.1x_{j}\left(t-1\right)+\overset{2}{\underset{j=1}{⋁}}0.02x_{j}\left(t-1\right)+0.01\;{\text{cos}}^{2}\;\left(\sqrt{17}t\right)]dt\\ & & +0.01\;\text{cos}\;\left(\sqrt{3}t\right)dw\left(t\right),\\ dx_{2}\left(t\right) & = & [-0.2x_{2}\left(t\right)+0.02\;\text{cos}\;\left(\sqrt{5}t\right)\;\text{cos}\;\left(x_{2}\left(t\right)\right)+0.03\;\text{cos}\;\left(\sqrt{2}t\right)\;\text{sin}\;\left(x_{1}\left(t-1\right)\right)\\ & & +\overset{2}{\underset{j=1}{⋀}}0.04x_{j}\left(t-1\right)+\overset{2}{\underset{j=1}{⋁}}0.2x_{j}\left(t-1\right)-0.02\left|\;\text{sin}\;\left(\sqrt{33}t\right)\right|]dt\\ & & +0.01\;\text{sin}\;\left(\sqrt{2}t\right)dw\left(t\right),\;\forall t\in R. \end{array}\right. \end{array}$$ (32)

**(1) Semi-discrete model**: base on model (32), we obtain the following semi-discrete model by using the semi-discretization technique:

$$\begin{array}{c} \left\{ \begin{array}{ccc} x_{1}\left(k+1\right) & = & e^{-1}x_{1}\left(k\right)+\left(1-e^{-1}\right)[0.01\;\text{sin}\;\left(\sqrt{5}k\right)\;\text{sin}\;\left(x_{1}\left(k\right)\right)\\ & & +0.05\;\text{sin}\;\left(\sqrt{7}k\right)\;\text{cos}\;\left(x_{2}\left(k-1\right)\right)+\overset{2}{\underset{j=1}{⋀}}0.1x_{j}\left(k-1\right)\\ & & +\overset{2}{\underset{j=1}{⋁}}0.02x_{j}\left(k-1\right)+0.01\;\text{cos}\;\left(\sqrt{3}k\right)\Delta w\left(k\right)+0.01\;{\text{cos}}^{2}\;\left(\sqrt{17}k\right)],\\ x_{2}\left(k+1\right) & = & e^{-0.2}x_{2}\left(k\right)+\frac{1-e^{-0.2}}{0.2}[0.02\;\text{cos}\;\left(\sqrt{5}k\right)\;\text{cos}\;\left(x_{2}\left(k\right)\right)\\ & & +0.03\;\text{cos}\;\left(\sqrt{2}k\right)\;\text{sin}\;\left(x_{1}\left(k-1\right)\right)+\overset{2}{\underset{j=1}{⋀}}0.04x_{j}\left(k-1\right)\\ & & +\overset{2}{\underset{j=1}{⋁}}0.2x_{j}\left(k-1\right)+0.01\;\text{sin}\;\left(\sqrt{2}k\right)\Delta w\left(k\right)-0.02\left|\;\text{sin}\;\left(\sqrt{33}k\right)\right|], \end{array}\right. \end{array}$$ (33)

where k∈Z.

**(2) Discrete model formulated by Euler scheme**: base on model (32), we obtain the following discrete-time model by using Euler method:

$$\begin{array}{c} \left\{ \begin{array}{ccc} x_{1}\left(k+1\right) & = & 0.01\;\text{sin}\;\left(\sqrt{5}k\right)\;\text{sin}\;\left(x_{1}\left(k\right)\right)+0.05\;\text{sin}\;\left(\sqrt{7}k\right)\;\text{cos}\;\left(x_{2}\left(k-1\right)\right)\\ & & +\overset{2}{\underset{j=1}{⋀}}0.1x_{j}\left(k-1\right)+\overset{2}{\underset{j=1}{⋁}}0.02x_{j}\left(k-1\right)\\ & & +0.01\;\text{cos}\;\left(\sqrt{3}k\right)\Delta w\left(k\right)+0.01\;{\text{cos}}^{2}\;\left(\sqrt{17}k\right),\\ x_{2}\left(k+1\right) & = & 0.8x_{2}\left(k\right)+0.02\;\text{cos}\;\left(\sqrt{5}k\right)\;\text{cos}\;\left(x_{2}\left(k\right)\right)+0.03\;\text{cos}\;\left(\sqrt{2}k\right)\;\text{sin}\;\left(x_{1}\left(k-1\right)\right)\\ & & +\overset{2}{\underset{j=1}{⋀}}0.04x_{j}\left(k-1\right)+\overset{2}{\underset{j=1}{⋁}}0.2x_{j}\left(k-1\right)\\ & & +0.01\;\text{sin}\;\left(\sqrt{2}k\right)\Delta w\left(k\right)-0.02\left|\;\text{sin}\;\left(\sqrt{33}k\right)\right|, \end{array}\right. \end{array}$$ (34)

where k∈Z.

**Fig 1:**
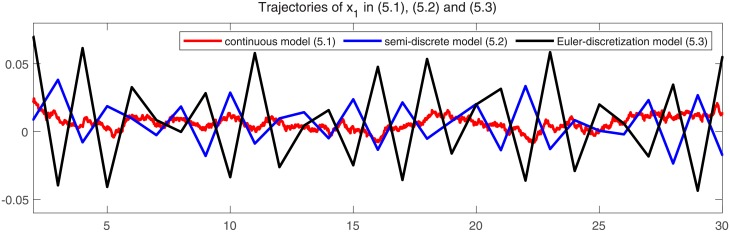
Trajectories of state variable *x*_1_ in models (32), (33) and (34), respectively.

**Fig 2:**
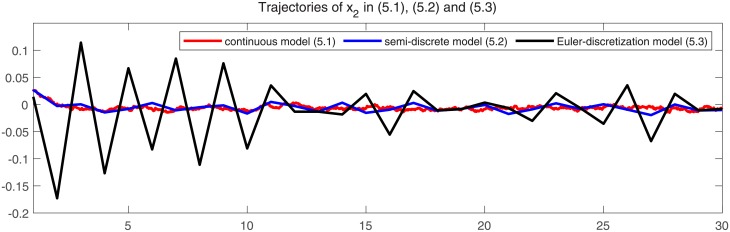
Trajectories of state variable *x*_2_ in models (32), (33) and (34), respectively.

In Figs 1 and 2, we give two plots of numerical solutions which are produced by continuous-time model (32), semi-discrete model (33) and Euler-discretization model (34), respectively. Compared with Euler-discretization model (34), semi-discrete model (33) gives a more accurate characterization for continuous-time model (32).

**Remark 4**. In literature [43, 44], the authors discussed the dynamics of periodic solutions of discrete-time neural networks formulated by Euler scheme. From the above discussion, semi-discrete stochastic system (5) gives a more accurate and realistic formulation for studying the dynamics of discrete-time neural networks. In a way, the work of this paper complements and improves some corresponding results in [43, 44].

Corresponding to system (5), we have $\underline{a}=1$, $\bar{a}=2$, $L_{i}^{f}=L_{i}^{g}=L_{i}^{\sigma }=1$, ${\bar{b}}_{ij}=0.02$, ${\bar{c}}_{ij}=0.05$, *α*_11_ = *α*_12_ = 0.1, *β*_11_ = *β*_12_ = 0.02, *α*_21_ = *α*_22_ = 0.04, *β*_21_ = *β*_22_ = 0.2, ${\bar{d}}_{ij}=0.01$, *i*, *j* = 1, 2.

Taking *p* = 2, by simple calculation,

$$\begin{array}{c} C_{2}^{1/2}=2,\;D^{*}\approx 0.74,\;K^{*}\approx 0.02,\;r_{4}\approx 0.85<1. \end{array}$$

According to Theorems 1 and 2, system (32) admits a square-mean almost periodic sequence solution, which is square-mean globally exponentially stable.

**Fig 3:**
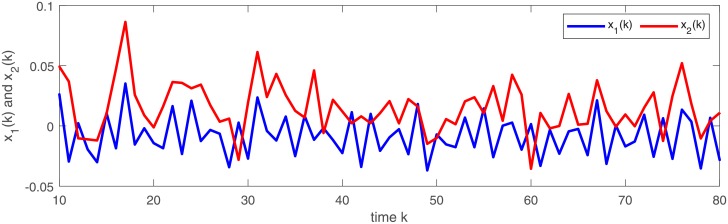
Square-mean almost periodicity of state variables (*x*_1_, *x*_2_)^*T*^ in model (33).

**Fig 4:**
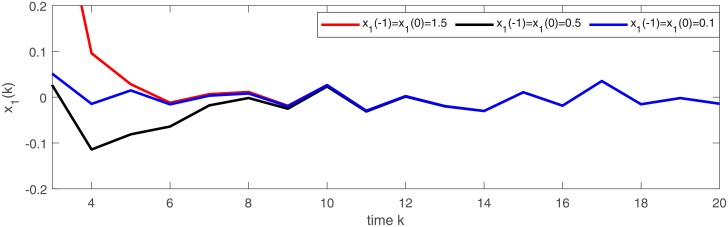
Square-mean global exponential stability of state variable *x*_1_ of model (33).

**Fig 5:**
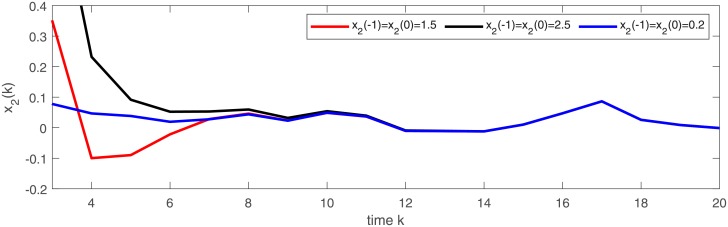
Square-mean global exponential stability of state variable *x*_2_ of model (33).

Fig 3 depicts a numerical solution (*x*_1_, *x*_2_) of semi-discrete stochastic model (33). Observe that the trajectories of (*x*_1_, *x*_2_) demonstrate almost periodic oscillations. Figs 4 and 5 display three numerical solutions of semi-discrete stochastic model (33) at different initial values (1.5, 1.5), (0.5, 2.5) and (0.1, 0.2), respectively. They are shown that semi-discrete stochastic model (33) is square-mean globally exponentially stable.

**Fig 6:**
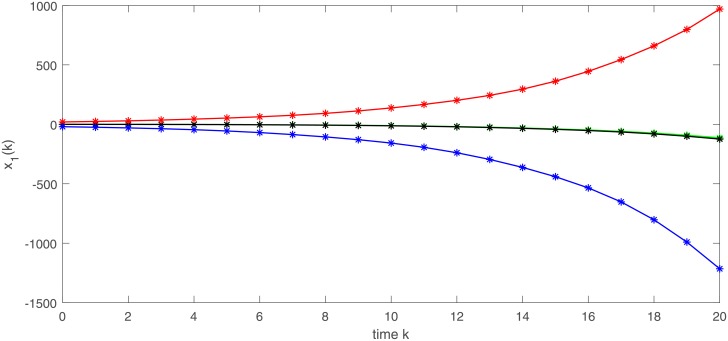
Exponential instability of state variable *x*_1_ of system (36).

**Fig 7:**
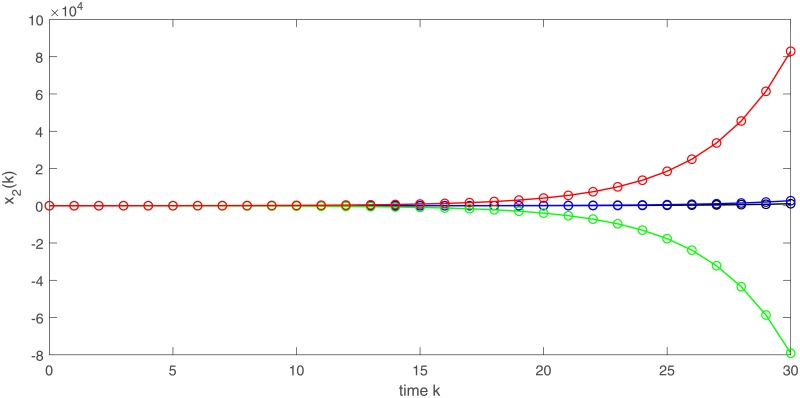
Exponential instability of state variable *x*_2_ of system (36).

**Example 2**. Considering the following deterministic cellular neural networks:

$$\begin{array}{c} \left\{ \begin{array}{ccc} {\dot{x}}_{1}\left(t\right) & = & 0.2x_{1}\left(t\right)+0.01\;\text{cos}\;t\left|x_{1}\left(t\right)\right|+0.01\;\text{sin}\;t\;\text{sin}\;x_{2}\left(t\right)+\;\text{sin}\;\left(0.1t\right),\\ {\dot{x}}_{2}\left(t\right) & = & 0.3x_{2}\left(t\right)+0.02\;\text{sin}\;t\left|x_{1}\left(t\right)\right|+0.01\;\text{cos}\;\left(\sqrt{5}t\right)\;\text{sin}\;x_{2}\left(t\right)+\;\text{cos}\;t, \end{array}\right. \end{array}$$ (35)

where t∈R. The following semi-discrete model for system (35) is obtained:

$$\begin{array}{c} \left\{ \begin{array}{ccc} x_{1}\left(k+1\right) & = & e^{0.2}x_{1}\left(k\right)\\ & & -\frac{1-e^{0.2}}{0.2}[0.01\;\text{cos}\;k|x_{1}\left(k\right)|+0.01\;\text{sin}\;k\;\text{sin}\;x_{2}\left(k\right)+\;\text{sin}\;\left(0.1k\right)],\\ x_{2}\left(k+1\right) & = & e^{0.3}x_{2}\left(k\right)\\ & & -\frac{1-e^{0.3}}{0.3}[0.02\;\text{sin}\;k|x_{1}\left(k\right)|+0.01\;\text{cos}\;\left(\sqrt{5}k\right)\;\text{sin}\;x_{2}\left(k\right)+\;\text{cos}\;k], \end{array}\right. \end{array}$$ (36)

where k∈Z. Obviously, system (36) satisfies (*H*_6_) in Theorem 6. So system (36) is exponentially instable, see Figs 6 and 7.

**Fig 8:**
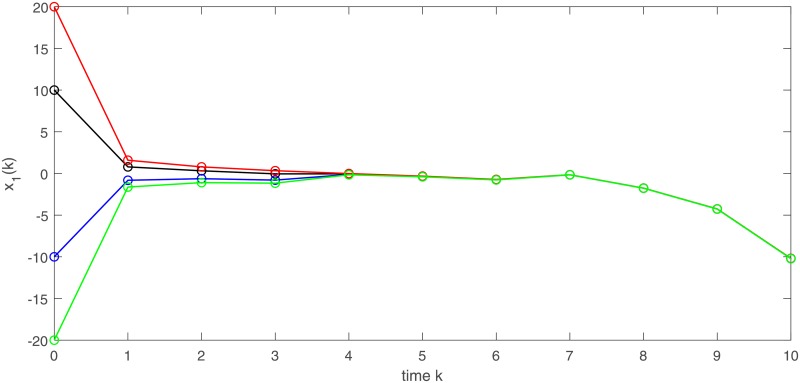
Exponential stability of state variable *x*_1_ of system (37).

**Fig 9:**
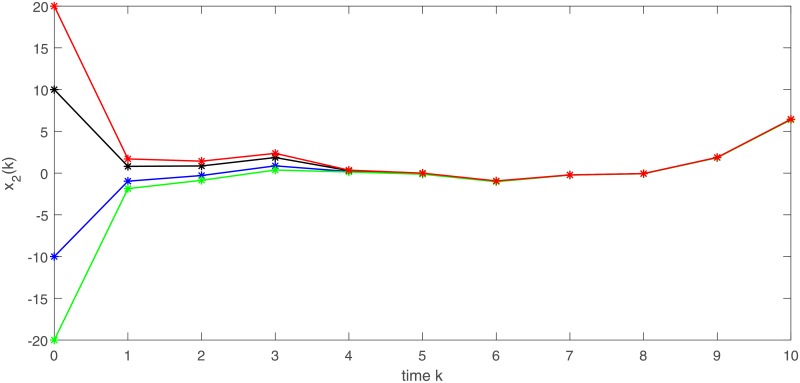
Exponential stability of state variable *x*_2_ of system (37).

Similar to the discussion as that in (SM), we consider a random perturbation in system (35) and a semi-discrete model with random perturbation is achieved as follows:

$$\begin{array}{c} \left\{ \begin{array}{ccc} x_{1}\left(k+1\right) & = & e^{p_{1}\left(k\right)}x_{1}\left(k\right)+\frac{\left(1-e^{0.2-0.5\kappa^{2}}\right)e^{\kappa \Delta w(k)}}{-0.2+0.5\kappa^{2}}[0.01\;\text{cos}\;k\left|x_{1}\left(k\right)\right|\\ & & +0.01\;\text{sin}\;k\;\text{sin}\;x_{2}\left(k\right)+\;\text{sin}\;\left(0.1k\right)],\\ x_{2}\left(k+1\right) & = & e^{p_{2}\left(k\right)}x_{2}\left(k\right)+\frac{\left(1-e^{0.3-0.5\kappa^{2}}\right)e^{\kappa \Delta w(k)}}{-0.3+0.5\kappa^{2}}[0.02\;\text{sin}\;k\left|x_{1}\left(k\right)\right|\\ & & +0.01\;\text{cos}\;\left(\sqrt{5}k\right)\;\text{sin}\;x_{2}\left(k\right)+\;\text{cos}\;k], \end{array}\right. \end{array}$$ (37)

where *p*_1_(*k*) = 0.2 − 0.5*κ*^2^ + *κ*Δ*w*(*k*), *p*_2_(*k*) = 0.3 − 0.5*κ*^2^ + *κ*Δ*w*(*k*), Δ*w*(*k*) = *w*(*k* + 1) − *w*(*k*), k∈Z. Here we choose stochastic disturbance coefficient *κ* = 1. It easily calculate (*H*_4_) in Theorem 4 is satisfied. Then system (37) is exponentially stable, see Figs 8 and 9. By Theorem 7, system (37) is a stochastic stabilization system of system (36).

## Conclusions and future works

In this paper, we formulate a discrete analogue of cellular neural networks with stochastic perturbations and fuzzy operations by using semi-discretization technique. The existence of *p*-th mean almost periodic sequence solutions and *p*-th moment global exponential stability for the above models are investigated with the help of Krasnoselskii’s fixed point theorem and stochastic theory. The main results obtained in this paper are completely new and the methods used in this paper provide a possible technique to study *p*-th mean almost periodic sequence solution and *p*-th moment global exponential stability of semi-discrete models with stochastic perturbations and fuzzy operations.

With a careful observation of Theorems 1 and 2, it is not difficult to discover that

1. *p* > 1 is crucial to the *p*-th mean almost periodicity and moment global exponential stability of system (5).
2. From Example 2, stochastic disturbance could be a useful method, which brings unstable system to be stable.
3. The time delays have no effect on the existence of *p*-th mean almost periodicity and *p*-th moment global exponential stability of system (5).

In the future, the following aspects can be explored further:

1. The methods used in this paper can be applied to study other types of neural networks, such as impulsive neural networks, high-order neural networks, neural networks on time scales, etc.
2. Other types of fuzzy neural networks could be investigated, such as Takagi-Sugeno fuzzy neural networks.
3. Other dynamic behaviours of system (5) should be further discussed.
4. The case of *p* ∈ (0, 1] could be further explored.
